# Engineering Intervertebral Disc Regeneration: Biomaterials, Cell Sources and Animal Models

**DOI:** 10.1111/cpr.70046

**Published:** 2025-05-19

**Authors:** Sidong Yang, Farhad Soheilmoghaddam, Peter Pivonka, Joan Li, Samuel Rudd, Trifanny Yeo, Ji Tu, Yibo Zhu, Justin J. Cooper‐White

**Affiliations:** ^1^ Tissue Engineering and Microfluidics Laboratory (TE&M) Australian Institute for Bioengineering and Nanotechnology (AIBN), The University of Queensland St Lucia Queensland Australia; ^2^ Department of Orthopaedic Surgery Hebei Medical University Third Hospital Shijiazhuang China; ^3^ Hebei International Joint Research Centre for Spine Diseases Shijiazhuang China; ^4^ School of Mechanical Medical & Process Engineering Queensland University of Technology Brisbane City Queensland Australia; ^5^ Faculty of Medicine The University of Queensland St Lucia Queensland Australia; ^6^ School of Chemical Engineering The University of Queensland St Lucia Queensland Australia; ^7^ Institute for Health Innovation & Technology (iHealthtech) National University of Singapore Singapore Singapore; ^8^ Spine Labs St. George & Sutherland Clinical School, University of New South Wales Sydney New South Wales Australia

**Keywords:** biomaterials, intervertebral disc degeneration, IVD regeneration, spine, stem cells, tissue engineering

## Abstract

Intervertebral disc (IVD) degeneration is an age‐related problem triggering chronic spinal issues, such as low back pain and IVD herniation. Standard surgical treatment for such spinal issues is the removal of the degenerated or herniated IVD and fusion of adjacent vertebrae to stabilise the joint and locally decompress the spinal cord and/or nerve roots to relieve pain. However, a key challenge of current surgical strategies is the increasing risk of adjacent segment degeneration due to the disruption of native biomechanics of the functional spinal unit, dominated by the loss of the IVD. In the past two decades, research has focused on developing a number of bioengineering approaches to repair and regenerate the IVD; in particular, tissue engineering of the IVD, using bioscaffolds and stem cells represents a promising area. This review highlights the current tissue engineering approaches utilising biomaterials, animal models and cell sources for IVD regeneration and discusses future opportunities.

## Graphical abstract

This review highlights the current tissue engineering approaches utilising biomaterials, animal models and cell sources for intervertebral disc regeneration and discusses future opportunities.

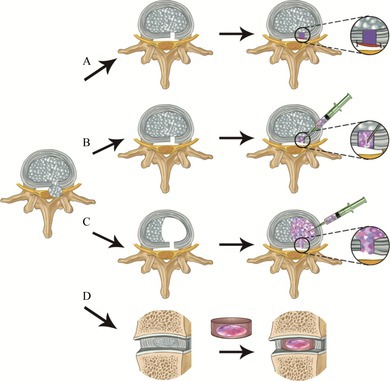

**Abbreviations**
ABNP: acellular bovine nucleus pulposus
ABS: acrylonitrile butadiene styrene
ACAN: aggrecan
ACs: articular chondrocytes
ADSCs: adipose‐derived stem cells
AF: annulus fibrosus
AFNP scaffold: silk protein scaffold for AF and fibrin/HA gel for NP
AFSCs: AF‐derived stem cells
AGE: advanced glycation end‐products
AIS: adolescent idiopathic scoliosis
ASD: adjacent segment degeneration
BDDE: 1,4‐butanediol diglycidyl ether
bFGF: basic fibroblast growth factor
BM: bone marrow
BM‐MSC: bone marrow‐derived mesenchymal stem cell
BMP: bone morphogenetic protein
CCGC scaffolds: one scaffold made of equine collagen, one of porcine collagen, one of gelatin and one of chitosan
CCL5: chemokine (C–C motif) ligand 5
CCS: collagen type II/chondroitin sulphate
CII/HyA/CS: collagen type II/hyaluronan/chondroitin‐6‐sulphate
Col I: collagen type I
Col II: collagen type II
CS: chondroitin sulphate
CTGF: connective tissue growth factor
DAFM: decellularised annulus fibrosus matrix
DAPS: disc‐like angle‐ply structures
DBP: demineralised bone particle
DCT: dextran, chitosan and teleostean
DEX: dexamethasone
dNPCS: decellularised nucleus pulposus‐based cell delivery system
ECM: extracellular matrix
EDC: 1‐ethyl‐3‐(3‐dimethylaminopropyl) carbodiimide HCL
ER: oestrogen receptor
FB/HA: fibrin/hyaluronic acid
FSU: functional spinal unit
GAG: glycosaminoglycan
GDF‐5: growth and differentiation factor‐5
GGMA: methacrylated gellan‐gum
H_2_O_2_
: hydrogen peroxide
HA: hyaluronic acid
hAMSCs: human amniotic mesenchymal stem cells
HRP: horseradish peroxidase
hUTC: human umbilical tissue‐derived cells
IL: interleukin
IPN: interpenetrating network
iPSCs: induced pluripotent stem cells
IVD: intervertebral disc
IVDD: intervertebral disc degeneration
LPS: lipopolysaccharide
LSS: leaf‐stack structural
MMP: matrix metalloproteinase
MPC: mesenchymal progenitor cell
mPCL/TCP: medical‐grade poly(epsilon‐caprolactone)/β‐tricalcium phosphate
MSC: mesenchymal stem cells
mTGase: microbial transglutaminase
NHS: n‐hydroxysuccinimide
NP: nucleus pulposus
OVX: ovariectomy
PCL: polycaprolactone
PDGF‐BB: platelet‐derived growth factor BB
PECUU: poly(ether carbonate urethane) urea
PEG: poly(ethylene glycol)
PEGDA: poly(ethylene glycol) diacrylate
PGA: polyglycolic acid
PLA: polylactic acid
PLGA: poly(l‐lactic‐*co*‐glycolic acid)
PLL: poly‐l‐lysine
PLLA: poly(l‐lactide)
PNIPAAm‐g‐CS: poly(*N*‐isopropylacrylamide)‐*graft*‐chondroitin sulphate
PPS: pentosan polysulphate
PTEN: phosphatase and tensin homologue deleted on chromosome 10
SA: sodium alginate
TDR: total disc replacement
TGF‐β: transforming growth factor‐β
TGF‐β1: transforming growth factor‐β1
TMHA: thiol‐modified hyaluronic acid
TMHA/EP: thiol‐modified hyaluronan elastin‐like polypeptide composite
TNF‐α: tumour necrosis factor‐α
UV: ultraviolet
WJ‐MSC: Wharton's Jelly‐derived mesenchymal stem cell

## 1 Introduction

Intervertebral disc (IVD) degeneration (IVDD), a key contributor to discogenic lower back pain (LBP) [1, 2, 3, 4, 5], is an age‐related remodelling process of the IVD caused by a reduction in proteoglycan content. This leads to decreased IVD height, sclerosis of the cartilage endplate and osteophyte formation [6, 7, 8]. The most common symptom caused by IVDD is chronic LBP, a major neurological disorder affecting more than 540 million people globally [9, 10], with a higher risk in females in the 40–69 years of age group, and a higher prevalence in high‐income countries compared with middle‐ to low‐income countries [11]. As a chronic and often debilitating condition, patients with IVDD‐associated symptoms generally lead a lower quality of life, translating to a substantial societal socioeconomic burden [12]. Nowadays, LBP has been the leading cause of disability worldwide, with the numbers expected to rise with an aging population [13, 14, 15].

Patients with IVDD are at risk of various severe degenerative disc diseases, including disc herniation, spinal canal stenosis, degenerative spondylolisthesis, and degenerative scoliosis [16, 17, 18]. Generally, these spinal diseases originate from the compression of the spinal cord and/or nerve roots, leading to leg pain, weakness and in severe cases, paralysis. The standard surgical treatment for such spinal issues is the removal of the herniated disc, with or without fusion of the adjacent vertebrae, to decompress the spinal canal. A major limitation of current surgeries is the inability of these procedures to replicate the biomechanical properties of the motion segment post‐surgery, leading to the acceleration of adjacent segment degeneration (ASD) and subsequent related spinal diseases [19]. Over the past number of decades, researchers have been seeking alternative approaches to repair and regenerate the IVD structure and restore its biomechanical function, and thus that of the functional spinal unit (FSU), to normal physiological levels [20].

Most recently, many of these attempts have taken a tissue engineering approach, combining biologically‐ or synthetically‐derived scaffolds, without or with patient‐derived IVD cells or stem cells, to regenerate the damaged tissues of the IVD, namely the nucleous pulposus (NP) and the annulus fibrosus (AF) [21, 22, 23, 24, 25, 26]. In this review, we summarised the literature published in the past two decades (2003–2023), aiming to provide a comprehensive overview of the tissue engineering approaches that have been applied to IVD regeneration and repair, covering the many different types of biomaterials, scaffolds and cell sources used, to provide a state‐of‐the‐art picture of where we, as a field, currently stand in terms of achieving this significant challenge, and highlighting opportunities for future investigations.

## 2 Intervertebral Disc

### 2.1 IVD Structure

**FIGURE 1:**
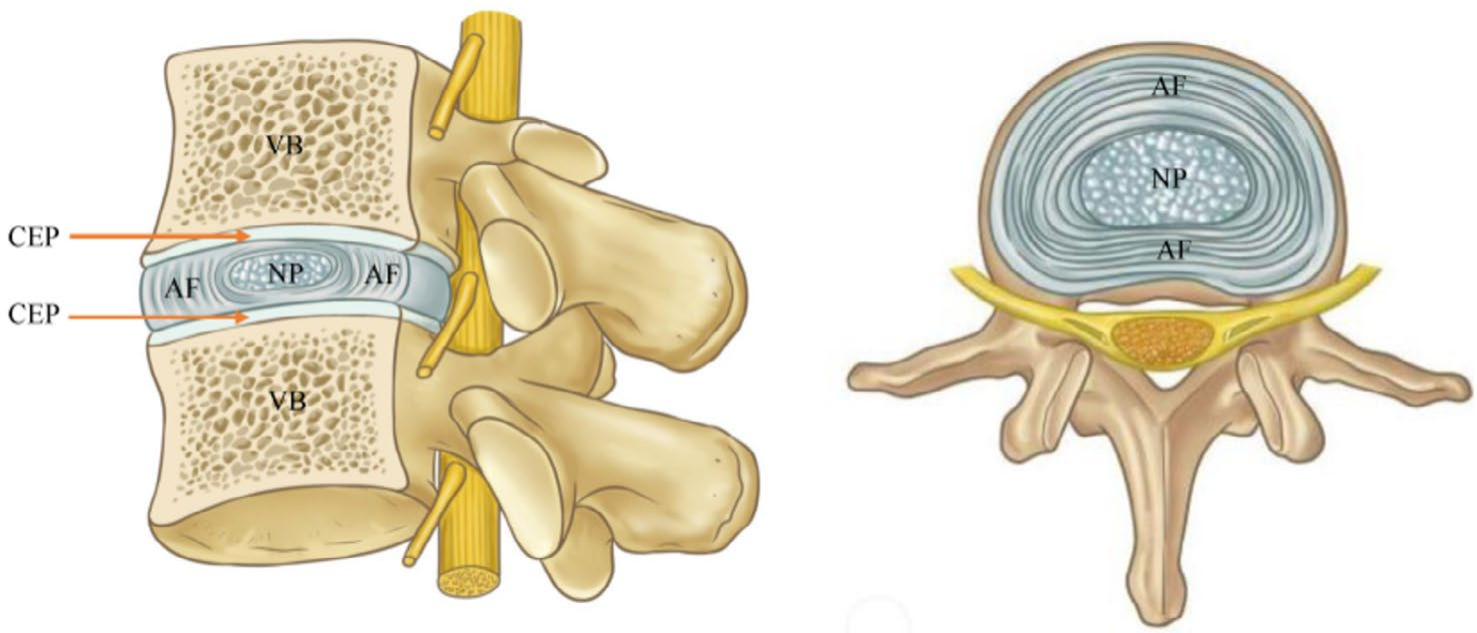
The structure of intervertebral disc. AF, annulus fibrosus; CEP, cartilage endplate; NP, nucleus pulposus; VB, vertebral body.

The spinal column supports the superior parts of the body and transmits loads between superior and inferior extremities [27]. As a connective structure of the spinal column, the IVDs absorb and disperse forces within the spine. IVDs are the largest avascular tissue in the human body, consisting of fibrocartilaginous tissue which connects two vertebral bodies (VB). The motion segment, which comprises two adjacent VBs and an IVD (i.e., the VB‐disc‐VB structure), is commonly referred to as a functional spinal unit (FSU). As shown in Figure 1, the structure of a normal human IVD can be distinctly divided into three parts: the AF, NP and cartilaginous endplates (CEP) [28, 29]. The outer AF is composed of fibroblast‐like cells and predominantly type I collagen. The soft, inner NP is derived from the notochord, unlike the AF and the CEP, which are derived from the mesoderm. NP cells are notochordal cells in the foetus, but by adulthood the notochordal cells are completely replaced by chondrocyte‐like cells [30, 31]. NP cells are surrounded by extracellular matrix (ECM) which is comprised of various biomacromolecules, mainly proteoglycans and type II collagen, with higher water content [32, 33]. Finally, the CEPs which connect the IVD to the VBs, are porous tissues that allow nutrient diffusion toward the inner NP, thus working as part of a nutrient delivery system for the IVD [34].

### 2.2 NP Phenotype Markers

Within a tissue engineering framework, identifying NP‐specific markers is crucial to confirming the successful differentiation of stem cells to NP‐like cells. Although the IVD is comprised of three structures, IVDD has been mainly attributed to lesion of the NP. As a result, whether understanding degenerative cascades or considering cell‐based regenerative strategies, research into cellular phenotypes and their specific markers within the IVD has focused mostly on the NP. Even so, due to the complexity of a heterogenous population, a consensus in the research community regarding the NP cell phenotype remains to be achieved [35]. Such included phenotypic markers are typically standard chondrogenic genes, including aggrecan (ACAN), collagen type II alpha 1 (COL2α1) and sex determining region Y (SRY)‐box 9 (SOX‐9) [36, 37, 38, 39]. These markers have been verified to be expressed in healthy human NP cells and in mesenchymal stem cells (MSCs) transitioning to NP‐like cells [35, 40, 41, 42], however, they are also expressed in a multitude of other cell types, including AF cells and articular chondrocytes (ACs), suggesting the need for other more specific NP markers.

To date, a number of novel NP cell phenotypic markers have thankfully been identified. Many of these markers have been identified using numerous animal models of IVD degeneration and regeneration, although some studies have characterised marker expression in human IVD tissues. For example, Sakai et al. [43] performed a microarray analysis of beagle lumbar IVDs to compare the gene expression differences among AF cells, NP cells and ACs; their findings showed that mRNA levels of alpha‐2‐macroglobulin, keratin (KRT) 18 and neural cell adhesion molecule (CD56) were expressed more in NP than in both AF and ACs. Gilson et al. [44] reported KRT8 as an NP‐specific cell marker in bovine‐derived IVDs, whilst Sonic hedgehog (Shh), secreted from the notochord during development, was reported as critical in the formation of the NP in mice and thus suggested as an NP‐specific marker in this species [45].

For human, Power et al. [46] found that carbonic anhydrase XII (CA12) could be regarded as a NP‐specific marker in human IVDs, both in healthy and degenerative IVDs. Richardson et al. [47] found the following NP markers in human IVD tissues: forkhead box F1 (FOXF1), Paired box protein 1 (PAX‐1), KRT8/18, CA12 and the notochordal cell markers brachyury, galectin‐3 and CD24 in cells of the NP, irrespective of age or degeneration. In addition, FOXF1 and PAX‐1 have also been confirmed by other studies [48, 49]. In another study, CD24, KRT8, KRT18 and KRT19 were posed as notochord‐specific markers during early human IVD development [30]. N‐cadherin and KRT19 have been recommended as common NP‐specific markers in healthy NP cells by a number of studies, as they could be used to distinguish NP cells from AF cells, ACs and even degenerated NP cells [50, 51]. Rodrigues‐Pinto et al. [35] have summarised NP markers in a species‐specific classification, which included rat, dog, bovine and human. Among them, at least four markers have been identified in each species, and most of the markers in one species are different from those in the other species. Nevertheless, five markers have been found in at least two species, those being KRT8, KRT18, KRT19, N‐Cadherin and FOXF1, noting that only KRT8, KRT18 and KRT19 belong to the notochordal ontogeny [52]. Specifically, KRT18, KRT19 and FOXF1 have been identified in human NP cells. Hypoxia‐inducible factor (HIF)‐1α is also often regarded as a NP‐specific marker, which is differentially expressed in NP cells under hypoxic conditions [53, 54]. HIF‐1α is a key transcription factor that is expressed in NP cells and can be used as a phenotypic marker of NP cells. Abundant GAG and type II collagen were detected under hypoxic conditions, with a high level of HIF‐1α, which is helpful for IVD regeneration [53]. Additionally, HIF‐1α was found to negatively influence NP cell proliferation time in the degenerated discs [54]. In 2014, the Spine Research Interest Group at the Annual ORS Meeting in New Orleans recommended some NP phenotypic markers for healthy humans: stabilised expression of HIF‐1α, GLUT‐1, ACAN/COL2α1 ratio > 20, Shh, Brachyury, KRT18/19, CA12 and CD24 [55].

Beeravolu et al. [41] employed human MSC‐derived chondroprogenitor cells to repair damaged IVDs in a rabbit model. Human NP‐specific markers including ACAN, COL2α1, SOX‐9, KRT19 and FOXF1 were selected to verify the differentiation of chondroprogenitor cells to NP‐like cells. Minogue et al. [56] compared gene expression between human NP cells and ACs using complementary DNA microarray and qPCR. Twelve NP‐positive genes were found to be substantially expressed (more than 20 folds as compared with control). The same team found that the NP‐specific markers, PAX‐1 and FOXF1, were significantly expressed after chondrogenic differentiation of MSCs, indicating the formation of an NP‐like phenotype. The expression of NP‐specific markers has been shown to vary depending on the concentration of oxygen available to the cells during differentiation, that is, under hypoxic conditions (2% or 5% oxygen) compared with normoxic (20%) conditions. For example, under hypoxic conditions, the impact of growth and differentiation factor (GDF)‐5, a commonly utilised growth factor in differentiation media for NP cells, on the differentiation of human MSCs toward NP‐like cells was shown to be significantly improved through increased expression of KRT19, CA12 and FOXF1 [57]. Glucose transporter 1 (GLUT‐1), which is controlled by HIF‐1α and expressed in hypoxic conditions [58], has also been reported as an NP‐specific marker during glucose starvation [59].

### 2.3 Biomechanical Function of IVD

The principal function of the IVD is to transfer loads primarily from superior to inferior tissues. Interestingly, IVDs have often been likened to a shock absorber of the body [60]. As such, it is very important to understand the biomechanical characteristics of the IVD, particularly in designing surgical prostheses and implants. Generally, most biomechanical tests of human IVD have been performed based on testing an entire FSU [61]. However, these tests do not provide biomechanical information on the individual tissue structures of IVDs including NP, AF and CEP. The latter are obtained by performing mechanical tests of the individual structures.

In healthy human IVDs, the central NP maintains a high osmotic pressure (100 kPa) that generates pre‐strain in the outer AF [62, 63]. A recent study showed that the injury to the IVD under such a pre‐strain can alter boundary constraints and residual strain, leading to aberrant mechanosensing and promoting short‐term apoptosis and fibrotic cell phenotype formation, which in turn promotes the progression of IVDD [64]. This residual stress in degenerative discs was less than in healthy discs in all regions [65], because IVDD decreased the equilibrium stress versus strain considerably. Even at the same strain level, degenerated discs were not able to maintain the same tensile strength as healthy discs. Interestingly, a similar result can be found in the implantation of the total disc replacement (TDR), which led to a mean reduction in the principal stiffness coefficients of 100%, 91% and 98% in lateral bending, flexion‐extension and axial rotation, respectively, demonstrating that an unconstrained, low‐friction TDR does not effectively replicate the stiffness of the original IVD [66].

The biomechanical properties of the NP tissue have been characterised under confined compression [67], unconfined compression [68] and shear [69]. Depending on the underlying constitutive material assumption which commonly includes elastic solid, biphasic material or viscoelastic material, a wide range of material parameters have been reported in the literature. Yang and Kish performed confined cylindrical compression testing to estimate the NP bulk modulus and found that it was very high, that is, 1720 MPa and close to water (2072 MPa), confirming the high water content of healthy NP tissue [67]. On the other hand, in confined compression testing, the effective aggregate modulus was found to be 1.0 MPa in healthy human NP [67], while poroelastic models of NP tissue use a Young's modulus ranging from 4.5 to 1500 kPa [69]. Using a simple axial stress versus strain relation obtained from unconfined compression tests, Cloyd et al. estimated a Young's modulus from the ‘linear’ region as 5.4 kPa [68].

In the AF, the content of type I collagen gradually increases from the inner to the outer surface, while the content of type II collagen and aggrecan increase in the opposite direction [70, 71]. The anterior and outer zones of the AF are thus much stiffer than the posterior and inner zones [72, 73], in line with a lumbar disc usually protruding backwards (or laterally) in clinical settings. The elastic modulus of AF lamellae begins at 59 MPa and increases gradually to 136 MPa, from the inner to the outer zones [74, 75]. Under tension, the AF is stiffest along the fibre axis (average modulus = 183 MPa), and the circumferential direction (16 MPa) is the second, finally the radial, axial and cross fibre directions (where the average moduli were 0.3, 2.6 and 0.2 MPa, respectively) [61]. Studies focusing on the biomechanics of CEP are not extensive due to the great difficulty in harvesting this thin tissue. In the IVD structure, the interface between the VB and the CEP is quite weak while the CEP‐NP juncture is much stronger, which contributes to much of the CEP fragment formation in IVD herniation [76, 77]. Greater characterisation of the CEP is certainly needed and would be potentially helpful in understanding IVD failure mechanisms, as well as in designing intervertebral implants or grafts [78]. The estimated Young's modulus of the CEP was previously reported to be 23.8 MPa, although just like the NP [68], CEP was noted to exhibit nonlinear behaviours [61].

### 2.4 Pathogenesis of IVDD

**FIGURE 2:**
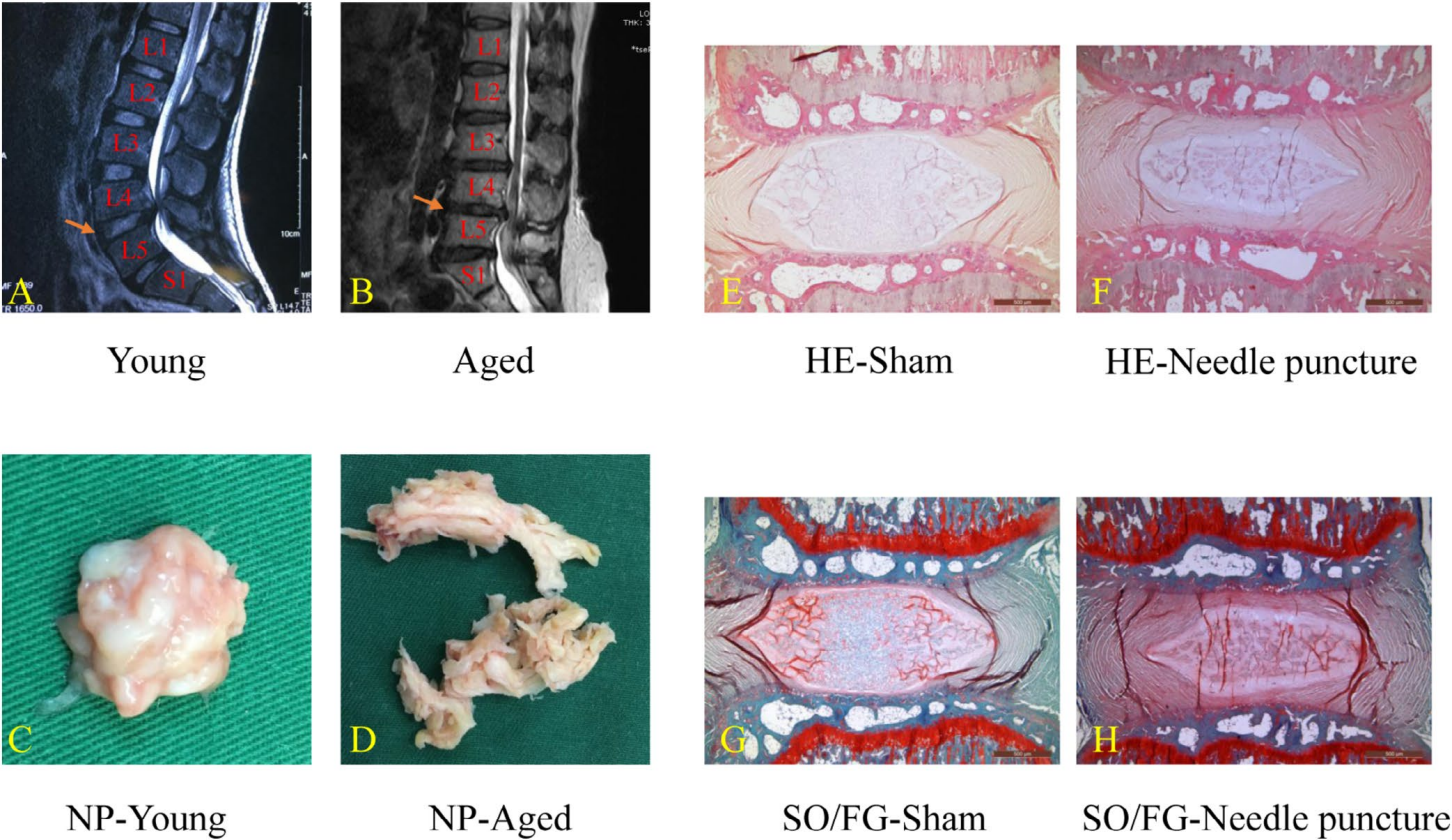
Comparisons between degenerative and nondegenerative intervertebral discs (IVD). (A–D) The young patient is 15 years old, male; the aged patient is 71 years old, female. Both patients suffered from L4 to L5 lumbar disc herniation. Nucleus pulposus (NP) of L4–L5 was removed intraoperatively to decompress the spinal canal. The young patient possesses a stronger MRI signal, higher intervertebral disc space, bigger lumbar lordosis and far more hydrated NP, compared with the aged patient. (E–H) A 21‐gauge needle was utilised to puncture a rat coccygeal IVD. Four weeks later, IVD degeneration was clearly observed with histological staining. By contrast, the sham group was handled without needle puncture. HE, Haematoxylin and eosin; SO/FG, safranin O/fast green.

While the pathological changes in IVDD are obvious in both NP morphology and histology, as shown in Figure 2, the pathogenesis of IVDD is complicated and remains to be completely elucidated. Cumulative evidence thus far indicates the following mechanisms: mechanical loading alterations, decreased nutrient supply to the IVD (through CEP calcification), NP cell senescence and aberrant apoptosis, excessive degradation of extracellular matrix (ECM) and increased inflammatory factors [33, 79, 80, 81, 82, 83]. Calcification of the CEP with age blocks the delivery of nutrients and oxygen exchange into the IVD, resulting in cell starvation, low oxygen and changes in pH (to acidic domains). The resulting poor microenvironment can further induce an increase in oxidative stress which leads to excessive cell death, as well as an imbalance of proteoglycans and type II collagen [84]. Furthermore, various inflammatory cytokines have been verified to be present in the progression of IVDD, such as interleukin (IL)‐1β, IL‐6 and tumour necrosis factor (TNF)‐α [85]. These cytokines can accelerate the degradation of proteoglycans and type II collagen by upregulating the expression of matrix metalloproteinase (MMP)‐1, 3, 13, ADAMTS‐4 (a disintegrin and metalloproteinase with thrombospondin motifs‐4) and ADAMTS‐5 [86, 87, 88].

A few etiological factors that are involved in the pathogenesis of IVDD are diabetes [89], smoking [90, 91, 92, 93, 94] and infection [95, 96, 97, 98]. Diabetes has been identified as one of the contributing factors for IVDD because the amyloid protein aggregation in diabetes has been found to accelerate the progression of IVDD [89]. Previous studies have reported that smoking has a negative impact on the IVD and thus contributes to the progression of IVDD in human beings [91, 93], rats [92, 94] and mice [90]. In addition, it is increasingly recognised that infection in IVD tissue could directly drive the progression of IVDD. In 2017, Shan et al. [96, 97] reported that the inoculation and incubation with *Propionibacterium acnes* in rabbit IVDs can result in mild inflammation, time‐dependent modic changes and signal intensity changes in MRI scan indicating obvious IVDD. One year later, the same research team performed a prospective clinical study investigating 66 cervical IVDs from 32 patients, finding that 10.6% of the samples were positive for coagulase‐negative Staphylococci and 3% were positive for *Propionibacterium acnes* [95]. The findings in a recent study further support the hypothesis that there might be a unique microbiome in human IVDs and that the dysbiosis can initiate the progression of IVDD [98].

Recently, more potential factors have been identified to contribute to IVDD progression. Cell cycle regulator p16 is known as a biomarker and an effector of aging. Che et al. [99] utilised a p16 knockout IVDD mouse model and found that p16 deletion can inhibit oxidative stress and promote NP cell cycle to finally ameliorate the progression of IVDD. Li et al. [100] reported that tissue inhibitor of metalloproteinase‐3 (TIMP‐3) might play an important part in the pathogenesis of IVDD because the deficiency of TIMP‐3 exists in degenerated discs and the overexpression of TIMP‐3 can significantly alleviate IVDD. Moreover, Mitofusin 2 was found to be expressed in human NP tissue, and the knockdown of Mitofusin 2 can aggravate mitochondrial dysfunction and cell death, accelerating IVDD, while the overexpression can significantly reverse IVDD progression [101]. Acid‐sensing ion channels exist in all IVD tissues, including NP, AF and CEP, and mediate IVDD via various pathways [102]. Based on a rabbit model, some researchers found that APOE‐knockout can cause NP cell loss and enhance inflammatory cytokines that damage the ECM, boosting IVDD formation [103]. In a recent study, Xi et al. [104] reported that phosphatase and tensin homologue deleted on chromosome 10 (PTEN) was overexpressed in degenerative NP, which could lead to an increase in NP cell apoptosis and senescence while decreasing the synthesis of ECM macromolecules, which promoted the progression of IVDD.

## 3 IVD Repair and Regeneration

**FIGURE 3:**
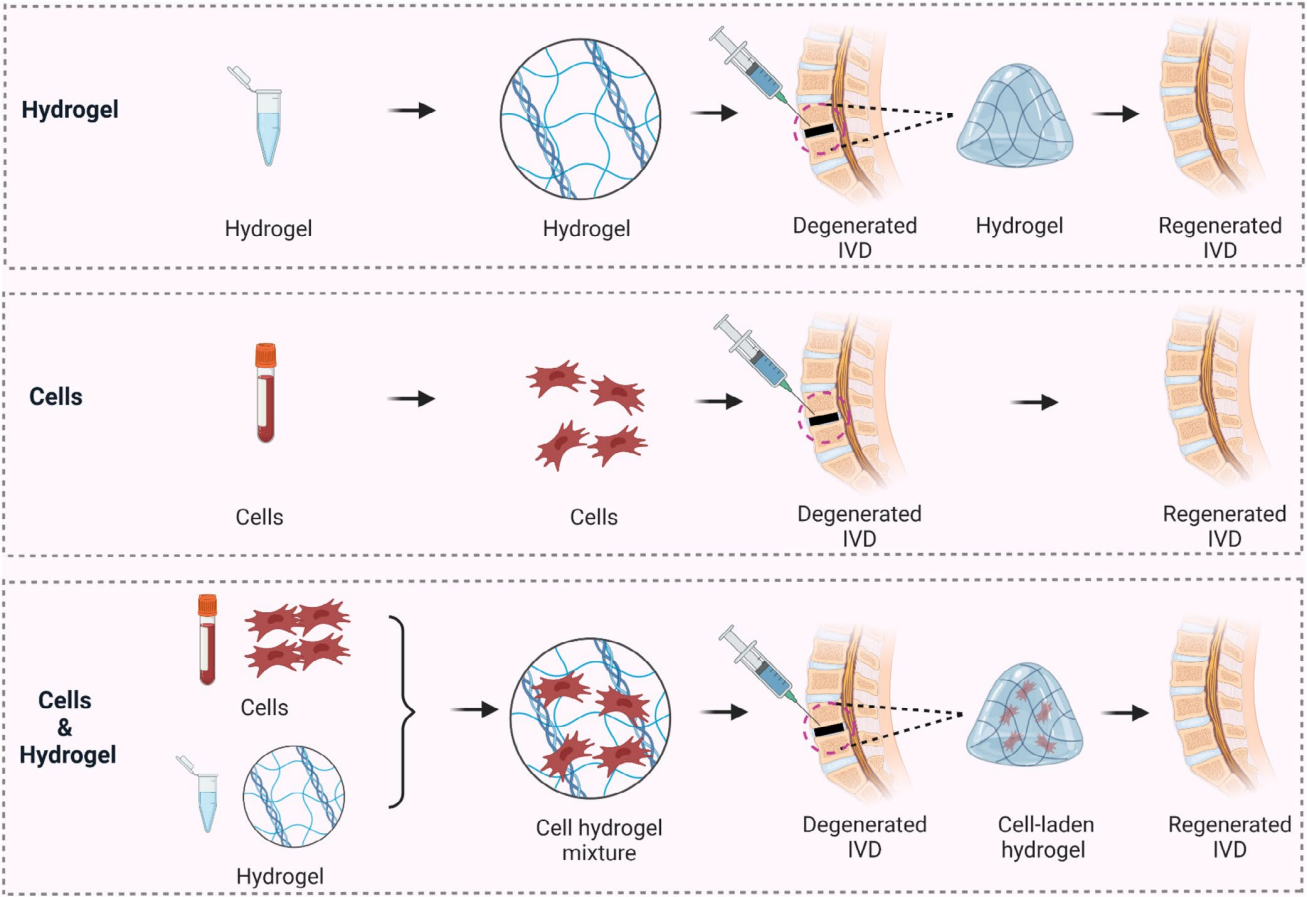
Injectable strategies for engineering intervertebral disc regeneration. Created with BioRender.com.

There are three main IVD repair strategies; the first is to directly supplement decreased or lost IVD cell stocks with exogenous cells, such as NP cells and stem cells [105]. The second approach is to stimulate native notochordal cells or NP cells to proliferate and produce more ECM components, including aggrecan and type II collagen, for self‐repair and functional regeneration of the IVD [106, 107]. In the last approach, injectable engineering strategies with or without cell encapsulation are used to regenerate IVD structures and restore their function [108, 109] (Figure 3). Importantly, IVD regeneration has been focused on the AF, NP or the entire IVD. This rarely includes the CEP, possibly because researchers have prioritised the AF and NP as more important structures.

Table 1 is a summary of tissue engineering studies targeting different IVD substructures for repair and regeneration. In essence, only 1 study focused on CEP regeneration, most studies covered the regeneration of NP tissue, around 25% of studies were on AF regeneration, with three studies targeting both AF and NP. Generally, it is well accepted that the preliminary problem of IVDD lies in NP dysfunction, and thus most researchers focus on the regeneration of the NP.

**TABLE 1:** Overview of bioengineering strategies used for IVD regeneration.

IVD substructure	Repair strategies	Studies: In vivo and in vitro	Total number of studies
AF	Using an AF repair patch. Nanocomposite hydrogel as a biological self‐gelling AF substitute. Using scaffold (or gel) encapsulating cells to treat AF defect.	[110, 111, 112, 113, 114, 115, 116, 117, 118, 119, 120, 121, 122, 123, 124, 125, 126, 127, 128, 129, 130, 131, 132, 133, 134, 135, 136, 137, 138, 139, 140, 141, 142, 143, 144, 145, 146, 147, 148]	39
NP	Bioscaffold/hydrogel with or without cells are directly implanted into the degenerated IVD for repair/regeneration of NP.	[36, 53, 149, 150, 151, 152, 153, 154, 155, 156, 157, 158, 159, 160, 161, 162, 163, 164, 165, 166, 167, 168, 169, 170, 171, 172, 173, 174, 175, 176, 177, 178, 179, 180, 181, 182, 183, 184, 185, 186, 187, 188, 189, 190, 191, 192, 193, 194, 195, 196, 197, 198, 199, 200, 201, 202, 203, 204, 205, 206, 207, 208, 209, 210, 211, 212, 213, 214, 215, 216, 217, 218, 219, 220, 221, 222, 223, 224, 225, 226, 227, 228, 229, 230, 231, 232, 233, 234, 235, 236, 237, 238, 239, 240, 241, 242, 243, 244, 245, 246, 247, 248, 249, 250, 251, 252, 253, 254, 255, 256, 257, 258, 259, 260, 261, 262, 263, 264, 265, 266, 267, 268, 269, 270, 271]	125
CEP	The regenerative CEP is a biomimetic three‐dimensional scaffold that supports nutrients and metabolites exchange between IVD and the milieu interior.	[272]	1
AF + NP	IVD scaffold consisting of an AF scaffold and a hydrogel‐based NP is implanted into the degenerated IVD to regenerate AF/NP. Hybrid bioadhesives combine an injectable glue to fill the NP cavity and a tough sealant to seal the AF defect simultaneously.	[273, 274, 275]	3

Abbreviations: AF, annulus fibrosus; CEP, cartilaginous endplate; IVD, intervertebral disc; NP, nucleus pulposus.

**FIGURE 4:**
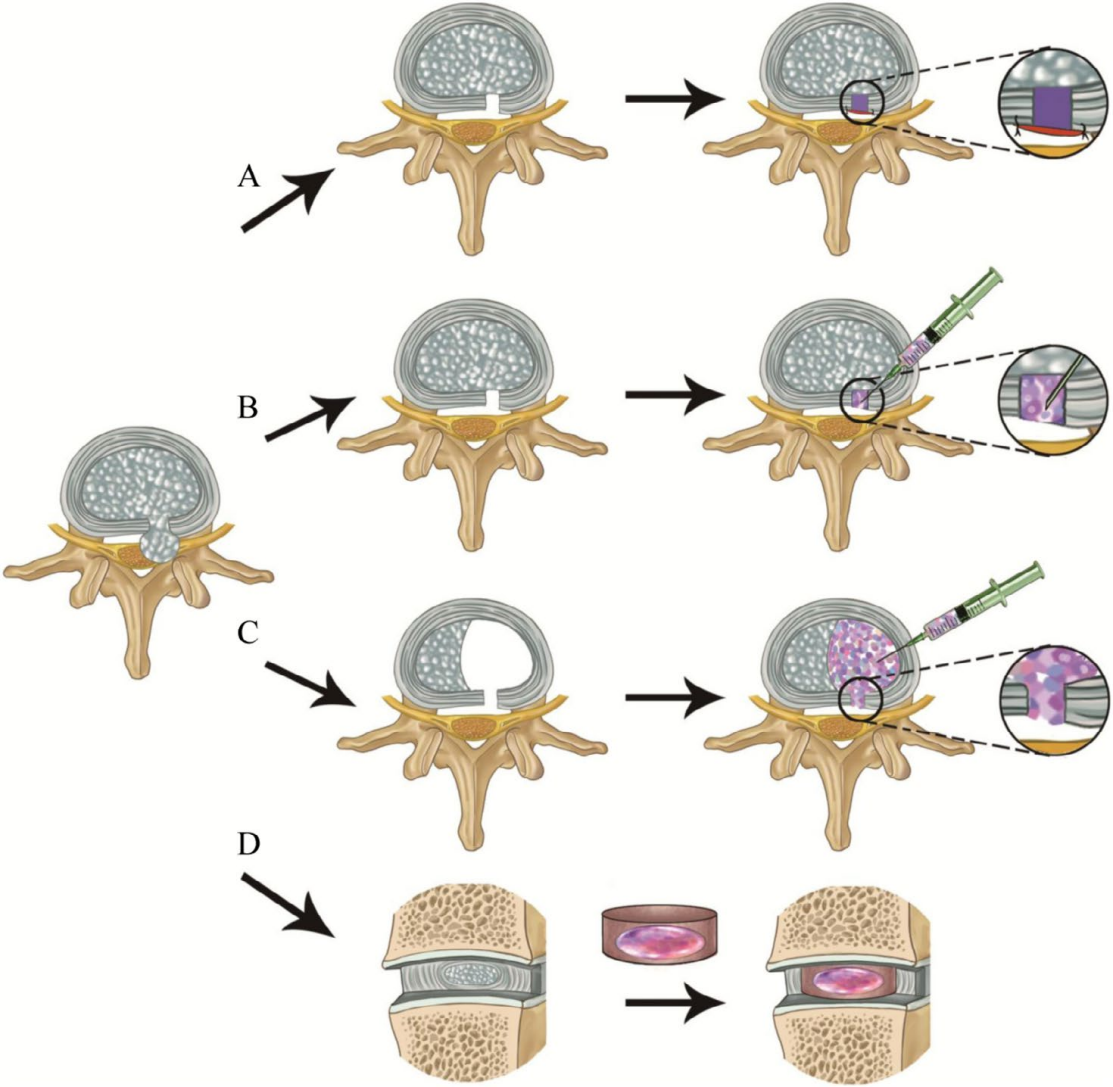
Schematic diagram of approaches used for engineering intervertebral disc regeneration. (A) AF repair using a bioengineering patch with a plug followed by proper suture to AF tissue. (B) AF repair by direct injection of hydrogel with or without encapsulating cells. (C) AF/NP repair by direct injection of hydrogel with or without encapsulating cells. (D) IVD regeneration by replacing the total IVD with a multiphasic bioscaffold loaded with stem cells or not. AF, annulus fibrosus; IVD, intervertebral disc; NP, nucleus pulposus.

**FIGURE 5:**
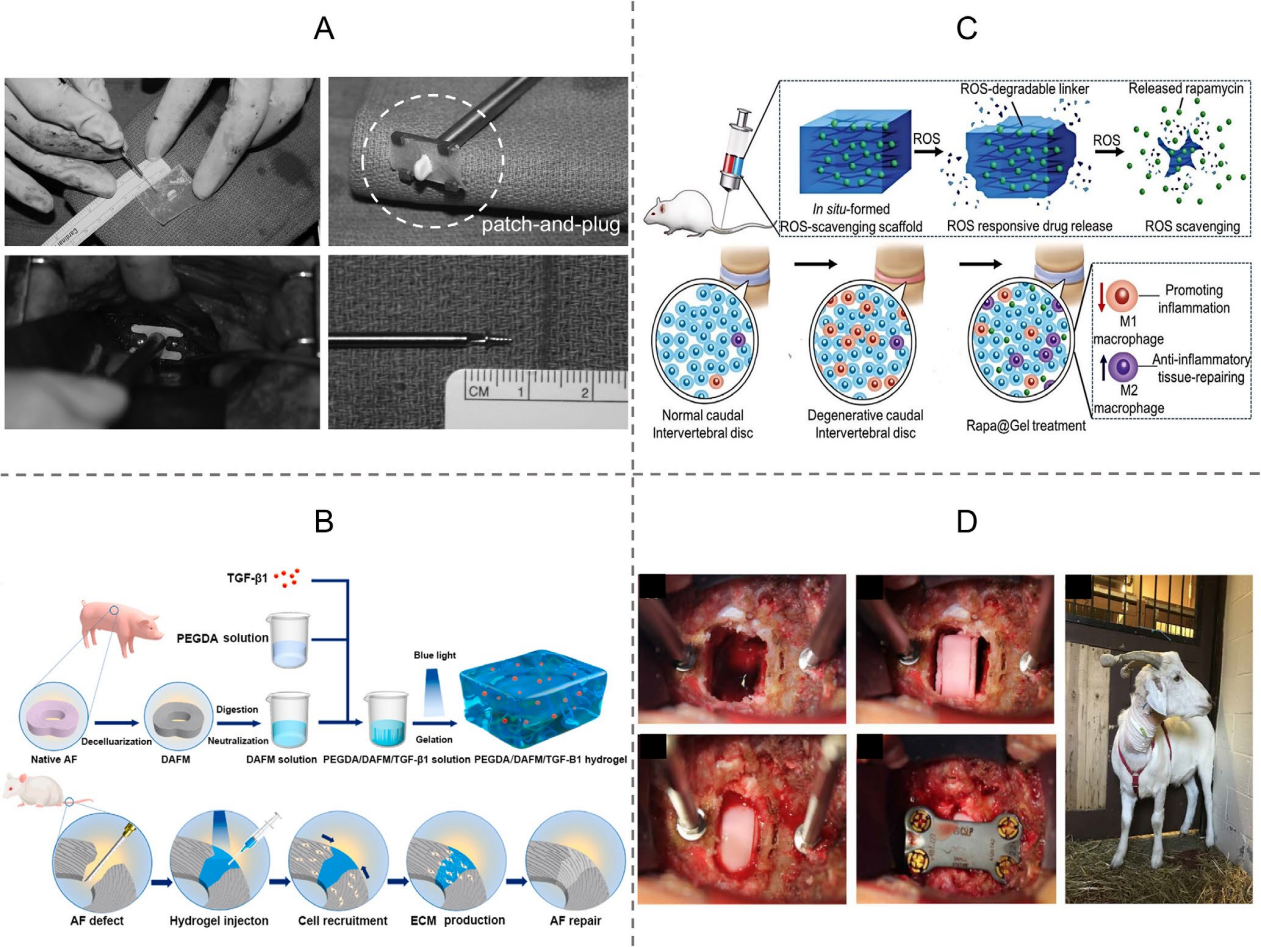
Representative studies of engineering IVD regeneration. (A) AF repair using a patch with a plug in a sheep model. Reproduced with permission from Ledet et al. [111]. Copyright 2009 Wolters Kluwer Health Inc. (B) TGF‐β1‐supplemented decellularised porcine AF matrix hydrogel promotes AF repair in a rat tail model [148]. Reproduced with permission under a creative commons attribution (CC BY 4.0) licence. Copyright 2023 authors. No changes were made to the reproduced figure. (C) IVD repair by direct injection of ROS‐scavenging hydrogel loaded with rapamycin in a rat model. Reproduced with permission from Bai et al. [239]. Copyright 2019 John Wiley and Sons. (D) IVD regeneration by replacement of a total IVD with a multiphasic bioscaffold loaded with stem cells in a goat model. Reproduced with permission from Gullbrand et al. [212]. Copyright 2018 The American Association for the Advancement of Science. AF, annulus fibrosus; IVD, intervertebral disc; ROS, reactive oxygen species; TGF‐β1, transforming growth factor beta 1.

As shown in Figures 4 and 5, among the approaches used for engineering repair and regeneration of the IVD structure, hydrogel injection is a popular approach utilised to repair the NP and AF, although engineered patch‐and‐plug approaches have also been utilised for repairing the native structure of the AF. Total IVD replacement using a multiphasic bioscaffold is a promising alternative being pursued for IVD regeneration. Biomaterials used for IVD repair, including hydrogel and other scaffolds, are summarised as follows.

### 3.1 Biomaterials

The development of tissue engineering‐based approaches to tissue repair is dependent on the development of biomaterials that are fit‐for‐purpose and that ideally encourage new tissue generation. A wide range of scaffolds made of various biomaterials have been investigated with or without cell (or stem cell) incorporation using different fabrication techniques. Table 2 summarises the studies utilising biomaterials targeting IVD tissue engineering published in the past two decades, most of which are polymeric (of natural, synthetic and natural/synthetic composite origins) in nature.

**TABLE 2:** Studies testing biomaterials for IVD repair/regeneration.

Reference	Year	Biomaterials	Crosslinking (crosslinker)	Inducing molecules	Cell type	Cell source	IVDD models
*Part I: Natural polymers*							
[191]	2023	Silk fibroin nanofiber reinforced alginate hydrogel	N/A	PRP	NPCs (in vitro)	IVDs, rat	Rat, coccygeal AF puncture
[273]	2023	Cellulose‐alginate hydrogel	N/A	GDF‐5	BM‐MSCs (in vitro)	Bone marrow, rats	Rat, TDR
[204]	2023	Collagen‐cryogel/HA	N/A	N/A	AD‐MSCs (in vitro)	Human, commercial	Rat, tail nucleotomy
[274]	2023	Alginate hydrogel/alginate‐polyacrylamide hydrogel	Ionically	N/A	NPCs (in vitro)	Human	N/A
[146]	2022	Acellular AF scaffold	N/A	N/A	hBMSCs	Unknown	N/A
[198]	2022	Alginate gel scaffold	Chemically (CaCl_2_?)	N/A	hBMSCs/hNPCs	Human	Sheep lumbar, discectomy
[143]	2022	Collagen‐based AF repair patch	Chemically (EDC/NHS)	N/A	AFCs/AD‐MSCs	Bovine AF/human	N/A
[200]	2022	Peptide hydrogel	N/A	N/A	NPCs	Bovine tails	N/A
[240]	2021	Chitosan nanocellulose hydrogel	N/A	N/A	AD‐MSCs	Sheep, autologous	Sheep lumbar, nucleotomy
[241]	2021	Collagen hydrogel	N/A	N/A	AD‐MSCs	Sheep, autologous	Sheep lumbar, nucleotomy
[193]	2021	Nanostructured gelatin colloidal hydrogels	Chemically (glutaraldehyde)	N/A	BM‐MSCs	Rabbit	Rabbit lumbar, AF puncture
[196]	2021	Decellularised nucleus pulposus hydrogel	Chemically (genipin)	N/A	AD‐MSCs	Rat	Rat tail, AF puncture
[242]	2021	Decellularised disc hydrogels	N/A	N/A	BM‐MSCs	Bone marrow, human	Rat tail, AF puncture
[201]	2021	HA hydrogel	Batroxobin	PRP	BM‐MSCs	Bone marrow, human	Bovine IVDs, nucleotomy (ex vivo)
[220]	2021	3D‐bioprinted gelatin/SA/HA scaffold	N/A	CTGF, TGF‐β3	BM‐MSCs	Rat	N/A
[239]	2020	PVA‐TSPBA hydrogel loaded with rapamycin	N/A	N/A	N/A	Rat	Rat tail, AF puncture
[188]	2020	IVD‐derived ECM hydrogel	N/A	Chondroitin sulphate	Nasal chondrocytes	Porcine nasal tissue	N/A
[186]	2020	GelMa microspheres	Photocrosslinking (UV light)	GDF‐5	AD‐MSCs	Rat	Rat tail, AF puncture
[145]	2020	Decellularised AF hydrogel	Chemically (genipin)	N/A	BM‐MSCs	Bone marrow, human	Rat tail, AF puncture
[122]	2020	Fibrin gel	N/A	CCL5	AFCs	Bovine tail IVDs	Sheep cervical IVDs, AF defect
[192]	2020	Ultra‐purified alginate gel	N/A	N/A	BM‐MSCs	Rabbit or human	Rabbit lumbar, partial discectomy
[199]	2020	Hyaluronan‐methylcellulose hydrogel	N/A	N/A	WJ‐MSCs	Human	Rat tail, AF puncture
[175]	2020	Low adhesive scaffold collagen	N/A	N/A	NPCs/AFCs	IVDs, human	Rat tail, nucleotomy
[217]	2019	Scaffold‐free tissue‐engineered construct (ECM)	N/A	N/A	AD‐MSCs	inguinal regions, rat	rat tail, total nucleotomy
[276]	2019	Self‐assembling peptide hybrid hydrogels	N/A	graphene oxide	N/A	N/A	N/A
[133]	2019	Cellulose nanofiber‐reinforced chitosan hydrogel	N/A	N/A	NIH/3T3 fibroblasts	NIH Swiss mouse embryo	Ex vivo, spine, pig
[130]	2019	HA scaffold	N/A	N/A	N/A	N/A	Bovin tail FSU, ex vivo
[219]	2019	Polymeric gelatin microsphere	Chemically (glutaraldehyde)	GDF‐5	NP‐like cells	Human iPSCs	Rat tail, AF puncture
[277]	2019	Chitosan hydrogel	Thermosensitive	N/A	NPCs	Coccygeal IVD, bovine	Human IVD, ex vivo
[176]	2019	3D‐RGD peptide‐modified polysaccharide hydrogel	N/A	N/A	NP‐MSCs	Tail IVDs, rats	Rat tail, AF puncture
[124]	2019	AF cell sheets	N/A	N/A	AFCs	Caudal IVD, SD rats	Rat tail, AF defect
[152]	2019	Biomimetic ABNP scaffold	Chemically (EDC/NHS, ethanol)	N/A	AD‐MSCs	Human, commercially	N/A
[151]	2019	Optimised decellularised NP scaffold (porcine)	N/A	N/A	hMSCs	Human, commercially	Rabbit, AF puncture
[155]	2018	Injectable dNPCS	Chemically (genipin)	N/A	AD‐MSCs	Groin, rabbit	Rabbit, AF puncture
[177]	2018	CCS composite hydrogel	Chemically (genipin)	N/A	AD‐MSCs	SD rat, commercially	SD rat, coccygeal AF puncture
[169]	2018	HA‐pNIPAM hydrogel	N/A	N/A	Autologous NPCs	Lumbar IVD, human	Bovine IVDs, induced by trypsin (ex vivo)
[222]	2018	BHNPs carrier	Chemically (EDC/NHS)	Human SDF‐1α	BM‐MSCs	Femoral shaft, SD rats	Rat tail IVD, AF puncture
[278]	2018	Carboxymethylcellulose hydrogels	Redox polymerised (APS, TEMED)	N/A	BM‐MSCs	Bone marrow, adult human	N/A
[279]	2018	Fibrin‐based hydrogels	Chemically (thrombin)	N/A	Chondrocytes	Knee joint, porcine	N/A
[183]	2017	Injectable triple‐IPN DCT hydrogel	N/A	N/A	MSCs and NPCs	BM, iliac crest, male goat	Male goat, chondroitinase ABC‐induced
[170]	2017	Gelatin‐poloxamer 407 cryogel	Chemically (EDC)	N/A	Dermal fibroblast cells	Human	N/A
[131, 137]	2017	Angle‐ply collagen AF patch	Chemically (EDC or glutaraldehyde)	N/A	AFCs	Caudal IVD, bovine	N/A
[272]	2017	HA‐CS‐COL II hydrogel	Chemically (EDC/NHS)	N/A	Chondrocytes	Costal cartilage, rabbit	N/A
[149]	2017	Chitosan‐HA hydrogel	Physically (β‐glycerophosphate)	Kartogenin	AD‐MSCs	Human	N/A
[127]	2017	Gelatin sponge transplant	N/A	Platelet‐rich plasma	BM‐MSCs	Iliac crest, goat	Goat, AF defect
[280]	2017	Ch‐β‐GP‐HA‐CS‐Col‐Ge‐FS hydrogel	Physically (β‐glycerophosphate)	N/A	NPCs	Lumbar disc, rabbit	N/A
[154]	2017	Small intestinal submucosa	N/A	N/A	NPCs	Lumbar disc, rabbit	Rabbit, AF puncture
[281]	2016	Type II collagen scaffold	N/A	N/A	AD‐MSCs	Rat, commercially	N/A
[282]	2016	Self‐assembling peptide hydrogel	N/A	N/A	NPCs	Bovine tails	N/A
[128]	2016	CS‐conjugated silk scaffold	Chemically (EDC or glutaraldehyde)	N/A	Articular chondrocytes	Goat cartilage	N/A
[283]	2016	TMHA hydrogel	Chemically (PEGSSDA)	PDGF‐BB	N/A	N/A	Rabbit, AF puncture
[284]	2016	Albumin/HA hydrogel	N/A	N/A	BM‐MSCs	Human	Bovine, ex vivo, IVD puncture/LPS/IL‐1β
[153]	2016	Biomimetic ABNP scaffold	Chemically (EDC/NHS)	N/A	hAMSCs	Amnion, human	N/A
[226]	2015	HA hydrogel	Chemically (butanediol diglycidyl ether)	N/A	WJ‐MSCs	Human umbilical cord	Rabbit, AF puncture + NP aspiration
[285]	2015	FB/HA hydrogel	Chemically (thrombin)	BMP‐2, BMP‐2/7 heterodimer	N/A	N/A	Goat, chondroitinase ABC‐induced
[172]	2015	Collagen‐LMW HA semi‐IPN	N/A	TGF‐β3	BM‐MSCs, nasal chondrocytes	Human	N/A
[134]	2015	Riboflavin crosslinked collagen gel	Photocrosslinking (blue light)	Riboflavin	N/A	N/A	Rat tail, AF puncture
[110]	2015	DBP gel (bovine femur)	N/A	N/A	AFCs	IVD, female rabbit	N/A
[237]	2015	Silk hydrogel	Chemically (EDC/NHS)	N/A	AD‐MSCs/NPCs	Human/bovine	N/A
[286]	2014	Porous silk fibroin scaffolds	N/A	N/A	NPCs	IVD, rabbit	N/A
[121]	2014	Alginate scaffolds	Chemically (EDC/NHS, AAD)	TGF‐β3	AFCs	Lumbar IVD, porcine	N/A
[287]	2014	EGC hydrogel	Chemically (EDC/NHS)	N/A	AD‐MSCs	Human, commercially	N/A
[181]	2014	HA‐albumin hydrogel	Chemically (HS‐PEG‐SH)	N/A	BM‐MSCs	Proximal tibia, porcine	Porcine, nucleotomy
[218]	2014	Gelatin/fibrin scaffold	N/A	PPS	MPCs	BM, iliac crest, sheep	Ovine, microdiscectomy
[136]	2014	Fibrin‐genipin adhesive hydrogel	Chemically (genipin)	N/A	N/A	N/A	Bovine caudal IVD (ex vivo)
[173]	2014	Self‐assembling peptide nanofiber hydrogel	Self‐crosslinking	BMP‐7	NPCs	Degenerated IVD, human	N/A
[288]	2014	Injectable triple‐IPN DCT hydrogel	N/A	N/A	BM‐MSCs	Femur/tibia, bovine	Cadaveric spine, nucleotomy
[180]	2014	HA hydrogel	N/A	N/A	BM‐MSCs	Femur/tibia, rabbit	Rabbit, AF puncture
[289]	2013	Fibrin hydrogel‐forming carrier	Chemically (thrombin)	N/A	hUTCs	Umbilical cord, human	Rabbit, AF puncture
[290]	2013	Oxi‐HAG‐ADH hydrogel	Chemically (EDC/NHS)	N/A	NPCs	IVD, rabbit	N/A
[194]	2013	Decellularised NP matrix	N/A	N/A	BM‐MSCs	Human, rabbit	Rabbit, AF puncture + NP aspiration
[215]	2012	HA hydrogel	N/A	N/A	BM‐MSCs, chondrocytes	Human tissues	Porcine, AF puncture
[171]	2012	Atelocollagen type I and type II scaffolds	Chemically (EDC)	TGF‐β1, BMP‐2	NPCs	Lumbar IVD, female rabbit	N/A
[150]	2012	Alginate and chitosan‐gelatin scaffolds	Chemically (glutaraldehyde)	N/A	NPCs	IVD, human	N/A
[291]	2012	Peptide nanofiber scaffold	Self‐crosslinking	N/A	NPCs	Lumbar IVD, male rabbit	N/A
[129]	2012	CS‐conjugated silk fibroin scaffold	Self‐crosslinking	N/A	Chondrocytes	Nasal cartilage, human	N/A
[232]	2012	Biphasic silk composite (AFNP) scaffold	N/A	N/A	AFCs, chondrocytes	IVD and articular cartilage, porcine	N/A
[292]	2012	Four types matrix scaffold (CCGC)	N/A	N/A	BM‐MSCs	BM, iliac crest and vertebrates, human	N/A
[293]	2011	Col II/HA scaffold	Chemically (4S‐StarPEG)	N/A	NPCs	Tail IVD, bovine	N/A
[294]	2011	C/G/GP hydrogel	Physically (thermosensitive)	Ferulic acid	NPCs	IVD, rabbit	N/A
[295]	2011	Silk fibrin/HA gel	Self‐crosslinking	N/A	Chondrocytes	Human, commercially	N/A
[179]	2011	CII/HyA/CS tri‐copolymer construct	Chemically (EDC/NHS)	N/A	NPCs	Thoracic/lumbar spine, rabbit	Rabbit, nucleotomy
[296]	2011	Acellular porcine NP‐ECM scaffold	N/A	N/A	AD‐MSCs	Human	N/A
[36]	2009	Hydrogel carrier (Puramatrix)	N/A	N/A	BM‐MSCs	BM, human	Porcine, AF puncture + NP aspiration
[297]	2009	Col I scaffold	N/A	N/A	N/A	N/A	N/A
[298]	2009	Alginate hydrogel	Photocrosslinking (UV light)	N/A	NPCs	Caudal IVDs, bovine	N/A
[223]	2009	HA hydrogel, CS hydrogel	Photocrosslinking (UV light)	N/A	N/A	N/A	Rabbit, AF puncture + NP aspiration
[224]	2009	PRP‐GHMs	Chemically (glutaraldehyde)	Platelet‐rich plasma	N/A	N/A	Rabbit, NP aspiration
[111]	2009	Small intestinal submucosa	N/A	N/A	N/A	N/A	Ovine, annulotomy
[299]	2008	Atelocollagen type II‐based scaffold	Enzymatically (mTGase)	N/A	NPCs	Tail IVDs, bovine	N/A
[236]	2008	Chitosan‐glycerophosphate hydrogel	Physically (thermosensitive)	N/A	BM‐MSCs	Femur, human	N/A
[225]	2007	Gelatin hydrogel microspheres	Chemically (glutaraldehyde)	Platelet‐rich plasma	N/A	N/A	Rabbit, NP aspiration
[205]	2006	Small intestinal submucosa scaffold	N/A	N/A	degenerative AF/NP cells	IVD, human	N/A
[227]	2006	COL II scaffold	N/A	N/A	BM‐MSCs	Iliac crest, rabbit	Rabbit, AF puncture + NP aspiration
[184]	2006	Col I scaffold	N/A	N/A	N/A	N/A	Bovine FSU, NP removal (in vitro)
[300]	2005	Gelatin/CS copolymer scaffold	Chemically (glutaraldehyde)	N/A	NPCs	Lumbar spine, AIS patients	N/A
[301]	2005	Gelatin/CS/HA copolymer scaffold	Chemically (glutaraldehyde)	N/A	NPCs	IVDs, human	N/A
[126]	2003	Atelocollagen honeycomb‐shaped scaffold	Photocrosslinking (UV light)	N/A	AFCs	IVDs, rabbit	Rabbit, laser discectomy
[125]	2003	Atelocollagen honeycomb‐shaped scaffold	Photocrosslinking (UV light)	N/A	AFCs	IVDs, rabbit	Rabbit, laser discectomy
[228]	2003	COL II scaffold	N/A	N/A	BM‐MSCs	Iliac crest, rabbit	Rabbit, AF puncture + NP aspiration
*Part II: Synthetic polymers*							
[275]	2022	3D printed PCL scaffold and GelMA	Physically (thermosensitive)	N/A	AFCs (in vitro)	IVDs, rat	Rat, TDR
[123]	2022	PECUU nanofiber scaffold	N/A	Fucoidan	AFCs (in vitro)	IVDs, rat	Rat, coccygeal AF defect
[189]	2020	PLLA nanofibrous spongy microspheres	N/A	Anti‐miR‐199a	BM‐MSCs	Femoral bone, rabbit	Rabbit, AF puncture
[185]	2020	PCL‐based LSS particles	N/A	TGF‐β3	BM‐MSCs	Human	Dog, lumbar, AF incision
[114]	2020	PCL‐PLLA electrospun fibre scaffold	N/A	N/A	N/A	N/A	N/A
[115]	2019	PCL‐PLLA electrospun fibre scaffold	N/A	N/A	Bovine AFCs	Tail IVDs, bovine	N/A
[168]	2019	GC/poly(EO‐*co*‐Gly) hydrogel	Physically (thermosensitive)	GDF‐5	Human iPSCs/rat NPCs	IVDs, rats (NPCs); iPSC (commercially)	Rat tail, AF puncture
[132]	2019	3D printed PCL scaffold	N/A	N/A	AFCs	Caudal IVD, bovine	N/A
[113]	2019	PCL, electrospun, aligned microfibers	N/A	N/A	N/A	N/A	Ovine, annulotomy of outer AF
[112]	2018	PCL nano/microfibrous scaffold	N/A	N/A	Autologous BM‐MSCs	Proximal tibia, porcine	Porcine, annulus defect
[158]	2017	PLGA scaffold	N/A	N/A	NPCs	IVD, female rabbit	N/A
[234]	2017	DAPS (PCL/PEO scaffold)	N/A	N/A	AF/NP cells	Caudal IVD, bovine	N/A
[162]	2017	L‐pNIPAAM‐*co*‐DMAc hydrogel	Physically (thermosensitive)	N/A	BM‐MSCs	Bone marrow, adult human	Bovine IVD tissue explants
[174]	2017	Polyplexes‐loaded PLGA nanospheres	N/A	N/A	NPCs	IVD, human	SD rat, IVD puncture
[159]	2017	PLGA scaffold	N/A	TGF‐β1	BM‐MSCs, NPCs	Human	Rabbit, nucleotomy
[142]	2016	Biodegradable PECUU scaffold	N/A	N/A	AFSCs	IVD, female rabbit	N/A
[233]	2015	Radiopaque PCL scaffold	N/A	N/A	BM‐MSCs in vitro	Proximal femur, bovine	Rat coccygeal spine, TDR
[156]	2015	3D‐printed ABS and PLA scaffolds	N/A	N/A	Chondrocytes and NPCs	Femur cartilage, caudal IVD, bovine	N/A
[135]	2015	Electrospun PECUU scaffold	N/A	N/A	AFSCs, BM‐MSCs	IVD and femur, rabbit	N/A
[238]	2015	PCL scaffold	N/A	N/A	AFCs	IVD, rabbit	N/A
[119]	2015	PTMC scaffold	Photocrosslinking (UV light)	N/A	BM‐MSCs	Human	Bovine organ culture annulotomy model
[231]	2014	PCL nanofibers	N/A	N/A	N/A	N/A	Rat caudal disc, TDR
[302]	2013	3D nanofibrous PLLA scaffold	N/A	N/A	AF/NP cells	Degenerated IVD, human	N/A
[182]	2012	TMHA/EP scaffold	Chemically (PEGDA)	N/A	N/A	N/A	Porcine, chondroitinase ABC‐induced
[116]	2012	PLLA scaffold	N/A	TGF‐β1	AFCs	Tail IVD, bovine	N/A
[163]	2012	Nanofibrous PLLA scaffold	N/A	N/A	NPCs	IVD, male rabbit	Athymic rats, nucleotomy
[303, 304]	2012	Nanostructured 3D PLGA construct	N/A	DEX and bFGF	BM‐MSCs	BM, SD rat	N/A
[305]	2011	mPCL/TCP scaffold	N/A	N/A	BM‐MSCs	Iliac crest, pig	Porcine, interbody fusion
[157]	2011	PGA scaffold	N/A	N/A	NPCs	IVD, human	N/A
[178]	2011	TMHA/EP scaffold	Chemically (PEGDA)	N/A	NPCs	IVD, human	Rabbit, AF puncture
[53]	2011	3D nanofibrous PLLA scaffold	N/A	TGF‐β1	BM‐MSCs	Femoral bone marrow, rabbit	N/A
[160]	2010	PLGA scaffold	N/A	N/A	NPCs	Lumbar IVD, beagle dog	Canine, nucleotomy
[140]	2008	BMG/PPCLM biphasic scaffold	N/A	N/A	Chondrocytes	Rib cartilage, rabbit	N/A
[209]	2007	PVA hydrogel	Self‐crosslinking	N/A	N/A	N/A	Rabbit, AF puncture
[139]	2007	MA‐POM scaffold	N/A	N/A	AFCs	Lumbar IVDs, rat	N/A
*Part III: Natural/Synthetic Composites*							
[190]	2023	Decellularised extracellular matrix gel	Physically (thermosensitive)	Vasorin	NPCs (in vitro)	IVDs, human	Rat, coccygeal AF puncture
[148]	2023	PEGDA/DAFM hydrogel	Photocrosslinking (blue light)	TGF‐β1	AFCs (in vitro)	IVDs, rat	Rat, AF defect model
[144]	2022	DAFM/PECUU‐blended fibrous scaffolds	N/A	N/A	AFSCs (in vitro)	Unknown	Rat, coccygeal AF defect
[195]	2022	HA‐BDDE/HA‐pNIPAM hydrogel	N/A	N/A	NPCs	Bovine IVDs	Bovine IVD, ex vivo
[197]	2022	Nanozyme‐functionalised hydrogel microsphere	Photocrosslinking (UV light)	N/A	NPCs	Tail IVD, rat	Rat, coccygeal AF puncture
[187]	2021	PNIPAAM‐*g*‐CS hydrogel	Physically (thermosensitive)	GDF‐6	AD‐MSCs	Human, commercially	Porcine IVD, ex vivo
[221]	2021	HA‐pNIPAM‐NH2 hydrogel	Thermoreversible	SDF‐1	BM‐MSCs	Femoral/tibial bone marrow, rat	Rat, coccygeal AF puncture
[118]	2021	PCL‐supported type‐I collagen patch	Chemically (EDC/NHS)	N/A	AFCs	Goat IVDs	N/A
[120]	2020	PU scaffold with COL I hydrogel	N/A	TGF‐β1	AFCs	Human IVDs	Bovine IVD, ex vivo, annulotomy
[306]	2020	PCL scaffold with Col‐HA‐chitosan hydrogel	Chemically (4S‐StarPEG)	N/A	hMSCs	Human, commercially	N/A
[216]	2019	PEAD:heparin:GDF‐5 delivery platform	N/A	N/A	AD‐MSCs	Human, commercially	Male SD rats, coccygeal AF puncture
[230]	2019	Alginate hydrogel‐PEGDA‐microcryogel	N/A	N/A	Autologous ADMSCs	Inguinal region, canine	L‐IVD, canine, NP aspiration
[212]	2018	PCL‐HA based eDAPS	N/A	N/A	AF/NP cells	Bovine IVD	Rat caudal IVD, goat cervical IVD, TDR
[138]	2018	GGMA‐nanocomposite hydrogel	Self‐crosslinking	N/A	AFCs	Tail IVD, calves	N/A
[117]	2017	Biphasic Col I‐PCL scaffold	N/A	N/A	AD‐MSCs	Human	N/A
[164]	2017	Carrageenan gel‐infused‐PCL scaffold	N/A	N/A	NIH 3T3 murine fibroblasts	Murine	N/A
[307]	2017	Silk fibroin PU hydrogel	N/A	N/A	N/A	N/A	N/A
[161]	2017	Dextran‐gelatin‐PEG hydrogel	Photocrosslinking (UV)	N/A	NPCs	IVD, male SD rat	Rat, AF puncture + NP aspiration
[229]	2016	pNIPAM‐HA hydrogel	Physically (thermoreversible)	N/A	N/A	N/A	Bovine, partial nucleotomy
[235]	2015	PNIPAAm‐g‐CS hydrogel	Chemically	N/A	HEK‐293 cells (in vitro)	Human	N/A
[167]	2015	Polysaccharide and polyamide hydrogel	Physically (thermoreversible)	N/A	hBM‐MSCs, autologous NPCs	Human, bovine IVD	Bovine IVD, papain‐induced (ex vivo)
[165]	2014	Resorbable PGA‐HA scaffold	N/A	N/A	N/A	N/A	Ovine, partial nucleotomy
[210]	2014	Gelatin PEG‐tyramine hydrogel	Enzymatically (HRP, H_2_O_2_)	Simvastatin	N/A	N/A	Rat, AF puncture
[206, 207]	2013/14	PEG‐HA based hydrogel	Enzymatically (HRP, H_2_O_2_)	PPS	MPCs	BM, human	N/A
[208]	2014	PEG‐HA hydrogel	Physically	N/A	AF/NP cells	Lumbar spine, pig	N/A
[166]	2014	PGA‐HA implant	N/A	N/A	N/A	N/A	Rabbit, partial nucleotomy
[308]	2013	PNIPAAm‐g‐CS hydrogels	Self‐crosslinking	N/A	HEK‐293 cells (in vitro)	Human embryonic kidney	N/A
[211]	2012	Serum albumin‐based PEG hydrogel	Chemically (SH‐PEG)	N/A	IVD cells	IVD tissues, human	N/A
[309, 310]	2012	GGMA hydrogels	Ionic‐/photo‐crosslinked	N/A	N/A	N/A	N/A
[213]	2010	PGA‐HA scaffold	N/A	N/A	N/A	N/A	Rabbit, partial nucleotomy
[311]	2009	PU‐fibrin scaffold	N/A	Fibrin	IVD cells	IVD tissues, human	N/A
[214]	2008	Cell‐free PGA‐HA implant	N/A	N/A	N/A	N/A	Rabbit, IVD resection

Abbreviations: 4S‐StarPEG, poly(ethylene glycol) ether tetrasuccinimidyl glutarate; AAD, adipic acid dihydrazide; ABNP, acellular bovine nucleus pulposus; ABS, acrylonitrile butadiene styrene; ACAN, aggrecan; AD‐MSCs, adipose‐derived mesenchymal stem cells; ADSCs, adipose‐derived stem cells; AF, annulus fibrosus; AFCs, annulus fibrosus cells; AFNP scaffold, silk protein scaffold for AF and fibrin/HA gel for NP; AFSCs, AF‐derived stem cells; AIS, adolescent idiopathic scoliosis; APS, ammonium persulphate; BDDE, 1,4‐butanediol diglycidyl ether; bFGF, basic fibroblast growth factor; BHNPs, albumin/heparin nanoparticles; BM, bone marrow; BMG/PPCLM scaffold, bone matrix gelatin/poly(polycaprolactone triol malate) scaffold; BM‐MSC, bone marrow‐derived mesenchymal stem cell; BMP, bone morphogenetic protein; C/G/GP hydrogel, chitosan/gelatin/glycerol phosphate hydrogel; CCGC scaffolds, one scaffold made of equine collagen, one of porcine collagen, one of gelatin and one of chitosan; CCL5, chemokine (C–C motif) ligand 5; CCS, collagen type II/chondroitin sulphate; Ch‐β‐GP‐HA‐CS‐Col‐Ge‐FS hydrogel, Chitosan‐β glycerophosphate‐hyaluronic acid, Chondroitin‐6‐sulphate, type II collagen, gelatin, fibroin silk hydrogel; CII/HyA/CS, collagen type II/hyaluronan/chondroitin‐6‐sulphate; Col I, collagen type I; Col II, collagen type II; collagen‐LMW HA semi‐IPN, collagen‐low molecular weight hyaluronic acid semi‐interpenetrating network; CS, chondroitin sulphate; CTGF, connective tissue growth factor; DAFM, Decellularised annulus fibrosus matrix; DAPS, disc‐like angle‐ply structures; DBP, demineralised bone particle; DCT, dextran, chitosan and teleostean; DEX, dexamethasone; dNPCS, decellularised nucleus pulposus‐based cell delivery system; ECM, extracellular matrix; eDAPS, endplate‐modified disc‐like angle ply structures; EDC, 1‐ethyl‐3‐(3‐dimethylaminopropyl) carbodiimide HCL; EDC/NHS, 1‐ethyl‐3‐(3‐dimethylaminopropyl) carbodiimide HCL/*n*‐hydroxysuccinimide; EGC hydrogel, elastin‐glycosaminoglycan‐collagen hydrogel; FB/HA, fibrin/hyaluronic acid; FSU, functional spinal unit; GAG, glycosaminoglycan; GDF‐5, growth and differentiation factor‐5; GGMA, methacrylated gellan‐gum; H_2_O_2_, hydrogen peroxide; HA, hyaluronic acid; hAMSCs, human amniotic mesenchymal stem cells; HEK‐293 cells, human embryonic kidney 293 cells; HRP, horseradish peroxidase; HS‐PEG‐SH, a specific thio‐polyethylene glycol; hUTC, human umbilical tissue‐derived cells; IL, interleukin; IPN, interpenetrating network; iPSCs, induced pluripotent stem cells; IVD, intervertebral disc; IVDD, intervertebral disc degeneration; L‐pNIPAM‐*co*‐DMAc hydrogel, laponite crosslinked poly(*N*‐isopropylacrylamide)‐*co*‐DMAc hydrogel; LPS, lipopolysaccharide; LSS, leaf‐stack structural; MA‐POM scaffold, malic acid‐based polyester poly(1,8‐octanediol malate) scaffold; MPC, mesenchymal progenitor cell; mPCL/TCP, medical‐grade poly(epsilon‐caprolactone)/β‐tricalcium phosphate; MSC, mesenchymal stem cells; mTGase, microbial transglutaminase; NHSS, *N*‐hydroxysulfosuccinimide; NP, nucleus pulposus; NPCs, nucleus pulposus cells; oxi‐HAG‐ADH hydrogel, oxidised hyaluronic acid‐gelatin‐adipic acid dihydrazide hydrogel; PCL, polycaprolactone; PDGF‐BB, platelet‐derived growth factor BB; PEAD, poly(ethylene argininylaspartate diglyceride); PECUU, poly(ether carbonate urethane) urea; PEG, poly(ethylene glycol); PEGDA, poly(ethylene glycol) diacrylate; PGA, polyglycolic acid; PLA, polylactic acid; PLGA, poly(l‐lactic‐*co*‐glycolic acid); PLL, poly‐l‐lysine; PLLA, poly(l‐lactide); PNIPAAm‐*g*‐CS, poly(*N*‐isopropylacrylamide)‐*graft*‐chondroitin sulphate; pNIPAM, poly(*N*‐isopropylacrylamide); PPS, pentosan polysulphate; PRP‐GHMs, platelet‐rich plasma‐impregnated gelatin hydrogel microspheres; PTMC scaffold, poly(trimethylene carbonate) scaffold; PU, polyurethane; PVA, poly(vinyl alcohol); SA, sodium alginate; SDF‐1α, stromal cell‐derived factor‐1α; TDR, total disc replacement; TEMED, tetramethylethylenediamine; TGF‐β1, transforming growth factor‐β1; TMHA, thiol‐modified hyaluronic acid; TMHA/EP, thiol‐modified hyaluronan elastin‐like polypeptide composite; UV, ultraviolet; WJ‐MSC, Wharton's Jelly‐derived mesenchymal stem cell.

#### 3.1.1 Natural Polymers

Natural polymers are extracted from natural sources including animal and plant tissues. Natural polymers utilised in IVD applications include polysaccharides, hyaluronic acid (HA), collagen, arginine, fibrin gel, gelatin, alginate, Matrigel and those present in a range of decellularised tissues [312, 313]. Poly(lactic acid) (PLA) and poly(glycolic acid) (PGA) have ever been reported as natural polymers [312], but they are regarded as synthetic polymers in most studies because they are synthesised from monomers, lactic acid and glycolic acid respectively. To date, natural polymers have been widely investigated in many studies for IVD tissue engineering, due to them offering significant advantages, including being biodegradable, biocompatible, renewable and vastly available [314].

As we see in Table 2, polysaccharides (cellulose, chitosan, alginate), hyaluronic acid (HA), ECM proteins (type I and type II collagen, fibrin, gelatin [denatured collagen], elastin), glycosaminoglycans (GAGs, including chondroitin sulphate [CS]) and decellularised tissues have all been investigated for the potential of attenuating IVDD progression—with HA being most widely tested. Almost all of the studies utilised the natural polymers to create bioscaffolds including injectable hydrogels, most of which were tested for biocompatibility by co‐culture with IVD cells or MSCs in vitro. In addition, some bioscaffolds were composed of only one polymer, such as HA hydrogels [284], but more composites were constituted of at least two types of natural polymers, such as chitosan‐HA hydrogel [149] and alginate‐chitosan‐gelatin scaffolds [150].

To date, decellularised matrix has been used in IVD engineering as a natural biomaterial. This decellularised tissue material is composed of ECM components, predominantly type I collagen and a small number of glycoproteins, as well as various growth factors [315, 316], making it beneficial to cell growth and proliferation and thus tissue repair and regeneration [317, 318, 319, 320]. One type of biomaterial that has attracted substantial attention is a decellularised NP matrix which mainly consists of collagen and proteoglycans [151, 152, 153, 154], mimicking the native NP architecture [155]. The decellularisation technique has developed to be a standard procedure which enables the researchers to harvest quality material [321, 322, 323]. A significant advantage of using a decellularised NP matrix is that most of the biological and biomechanical properties can be retained while the potential immunological issues caused by the implant can be minimised by removing the exogenous cellular components [154, 324]. There is, however, a downside, that being that the process of decellularising NP tissues can damage the intercellular substance and disrupt the ECM architecture and its composition, decreasing the biomechanical property of ECM and thus affecting the efficiency of the regeneration [325, 326]. Another type of biomaterial that differs from the decellularised matrix is fabricated by the demineralisation of bone tissue. Song et al. [110] demineralised bovine femur to produce a bone particulate gel which consists largely of type I collagen and contains various bioactive molecules, such as cytokines; it was found that such gels were helpful to promote AF cell proliferation and distribution and increase ECM production. Animal tissue‐derived biomaterials have also been assayed to repair IVD using small intestinal submucosa [111, 205], a naturally occurring biodegradable collagen‐based material [327]. When used as a resorbable graft to repair tissues, small intestinal submucosa can lead to efficacious repair and regeneration in many other tissues [328, 329, 330, 331, 332, 333, 334]. Results from two studies have indicated that small intestinal submucosa can effectively alleviate IVDD progression and promote IVD repair [111, 154, 205]. In an in vitro study, human degenerative AF and NP cells were seeded onto small intestine submucosa scaffolds; GAG was significantly increased over a 3‐month period, indicating the potential of small intestine submucosa scaffolds for IVD repair [205]. Later, in an in vivo sheep model, small intestinal submucosa was utilised for annular defect closure, finding that small intestinal submucosa can help maintain the hydration of IVD and lead to a 66% functional recovery compared with annulotomy alone levels [111]. Results from small intestinal submucosa are promising for IVD repair; however, few studies focused on small intestinal submucosa used for IVD repair.

Taken together, natural polymers used for IVD tissue regeneration have many merits including excellent biocompatibility, injectability, biodegradability, bioabsorbability and great availability; however, some non‐negligible drawbacks have limited their applications in clinical scenarios, such as poor mechanical properties, fragility and rapid dissolution [335, 336, 337, 338, 339, 340, 341, 342]. Natural polymers may thus require supplementation with other synthetic polymers, as discussed below, to develop optimal biomaterial formulations best suited for IVD tissue engineering.

#### 3.1.2 Synthetic Polymers

Synthetic polymers have some significant advantages when compared with many natural polymers in IVD tissue engineering applications due to them possessing, by design, tunable and selectable degradation mechanisms and rates, improved mechanical properties, microstructure control and processability and minimal immunological issues [243, 244, 245]. Controlled biodegradability is crucial for the use of tissue engineering bioscaffolds in IVD repair and regeneration. The degradation rate of the biomaterials should complement that of the rates of IVD tissue generation, with the optimal outcome being complete degradation of the bioscaffolds once the lost volume of IVD tissue has been successfully replaced with new tissue. Multiple intrinsic and extrinsic factors influence the biodegradation of synthetic polymers, including polymer hydrophilicity, crystallinity, composition and bond chemistry, and environmental temperature and pH. For many of the well‐used polymers, due to them being ester‐based, passive or acid‐catalysed hydrolysis is the predominant mechanism by which they degrade [246].

A wide range of commercially‐available synthetic biodegradable polymers, including PLA [156], PGA [157], polylactide‐glycolic acid (PLGA) [158, 159, 160], polyethylene glycol (PEG) [161, 206, 207, 208], polycaprolactone (PCL) [112, 113], poly(vinyl alcohol) (PVA) [209] and poly(*N*‐isopropylacrylamide) (PNIPAAm) [162], have been assessed for their utility in IVD tissue engineering applications. Polyurethane (PU), which can be biodegradable or non‐biodegradable due to the great versatility in tailoring its physical properties and biodegradation characteristics [247], has also been employed as a biodegradable material [248] or as a non‐biodegradable material for IVD engineering [249]. Of all of these polymer systems, PEG, PCL, PLA and PLGA are the most commonly used in IVD tissue engineering [244, 250]. These polymers are well characterised and have gained predicate FDA approval for use in humans (such as bone screws) [251]. PEG is a nonionic, hydrophilic, elastomeric polymer, widely used for biomedical applications, owing to its nontoxicity, non‐immunogenicity, resistance to protein absorption and high aqueous solubility [252] and having been used as a component of bioscaffolds for IVD tissue engineering [25, 253]. PCL, a biodegradable and non‐toxic aliphatic polyester, has been well utilised in many load‐bearing applications due to its significant intrinsic stiffness, variable crystallinity and extended degradation periods (of up to 4 years). However, its hydrophobicity can impact cell adhesion and infiltration [244, 254], a deficiency that some have overcome by blending with other degradable polyesters, such as PLA. For example, the blending and electrospinning of PLA with PCL to form a three‐dimensional annular scaffold, showing improved bioactivity when cultured with bovine AF cells, compared with PCL alone [114, 115]. There are in fact three forms of PLA, poly(l‐lactic acid) (PLLA), poly(d‐lactic acid) (PDLA) and poly(dl‐lactic acid) (PDLLA). Among these PLA subtypes, PLLA has been used most in IVD tissue engineering, either combined with other polymers [114, 115] or alone [53, 116, 163, 302].

PLGA, a linear copolymer combining PLA with PGA, is one of the most widely used synthetic polymers for fabricating bioscaffolds in tissue engineering applications [251, 255]. PLGA is easily broken down in vivo by hydrolysis into lactic acid and glycolic acid, which are physiologically metabolised in the tricarboxylic acid cycle [256]. The use of PLGA has a number of advantages beneficial to its application in IVD tissue engineering, including good biocompatibility [257], excellent tunability in terms of the lactide:glycolide ratio, molecular weight, functionalisation [258] and versatile processability [259].

#### 3.1.3 Natural/Synthetic Composites

Composites aimed at combining the advantageous properties offered by both natural and synthetic polymers have also attracted much recent attention in IVD tissue engineering [260], as Table 2 exemplifies. Of the synthetic polymer systems explored thus far, PEG [161, 206, 207, 208, 210, 211], PCL [117, 118, 164, 212, 306] and PGA [165, 166, 213, 214] are the most popular, most often being combined with HA and gelatin.

HA‐PEG and gelatin‐PEG hydrogels have seen significant exploration as potential composites for IVD repair. In studies by Frith et al. [206, 207], the potential of HA‐PEG composites as an injectable enzymatically‐crosslinked (horseradish peroxidase [HRP] and hydrogen peroxide [H_2_O_2_]) hydrogel of tunable gelation rate and mechanical strength matching that of IVD tissues was investigated. When loaded with mesenchymal precursor cells (MPCs), these hydrogels supported MPC proliferation and differentiation for up to 21 days of culture, with confirmed development of cartilage‐like tissues. They also showed that the addition of pentosan polysulphate (PPS), a GAG mimetic, to these hydrogels significantly upregulated the production of type II collagen, but most importantly, that when bound to the HA backbone in these HA‐PEG hydrogels, HA‐PPS provided significant advantage over the addition of soluble free PPS into the hydrogel [207]. Jeong et al. have also shown that HA‐PEG hydrogels (crosslinked by Michael‐type addition reaction of thiols to vinylsulfones) can promote proliferation of primary NP cells and AF cells, and that hydrogels made from lower‐molecular‐weight HA work better by encouraging greater cell proliferation and higher GAG deposition [208]. Additionally, PEG has also been explored in combination with gelatin for IVD repair [118, 208]. In 2014, Than et al. [210] utilised injectable gelatin‐PEG hydrogel (enzymatically‐crosslinked by HRP/H_2_O_2_) as a carrier to deliver simvastatin into rats' needle‐injured IVDs, finding that 5 mg/mL simvastatin in the gelatin‐PEG hydrogel carrier restored the injured IVDs 24 weeks after simvastatin delivery, with comparable radiographic and histologic features to non‐injured IVDs. Later, Gan et al. [161] used dextran, gelatin and PEG to form an interpenetrating network hydrogel derived by Schiff‐base reaction and photo‐crosslink. After encapsulating porcine NP cells, this hydrogel was implanted into porcine lumbar IVDs. This implant was found to facilitate the rehydration and regeneration of porcine degenerative IVDs as long as 12 weeks postoperatively.

Studies investigating blends of PCL and HA‐based hydrogels as IVD scaffolds have also produced encouraging outcomes, showing them to be biocompatible, injectable and displaying biomechanical properties (e.g., compressive modulus, viscoelasticity) similar to native IVDs [212, 306]. Gullbrand et al. [212] developed a composite construct aimed at mimicking the layered structure of the native IVD. The NP region of this scaffold was composed of a bovine NP cell‐seeded HA‐based hydrogel, while the AF region consisted of bovine AF cell‐seeded, concentric layers of aligned nanofibrous PCL. The customised construct was transplanted into athymic rat coccygeal IVDs. It was found that the compressive mechanical properties approached native values, and physiological loads allowed functional integration after 20 weeks implantation. These authors subsequently performed an in‐depth investigation using this customised construct but seeded with bone marrow‐derived allogenic MSCs and cultured for 13–15 weeks before implantation in a goat cervical disc replacement model. After 4 weeks, the implant was preserved within the goat cervical disc space and matrix distribution and content were maintained or slightly improved compared with pre‐implantation values. After 8 weeks, T2‐weighted MRI showed increased signal intensity in the IVDs compared with pre‐implantation values, and compressive mechanical properties increased to a level comparable to native cervical IVDs, demonstrating that this particular construct offered significant potential for clinical translation. Along similar lines, Gloria et al. [306] customised a multiphasic IVD scaffold consisting of both NP and AF structures, made from a HA hydrogel (seeded with human MSCs) and PCL, respectively. Rheological assessment of the HA‐based hydrogel confirmed that they were shear‐thinning and injectable. Mechanical testing confirmed that the compressive modulus and the shape of the stress–strain curve of the composite IVD scaffold were similar to those of native lumbar IVDs. In vitro culture of the seeded construct revealed that the human MSCs remained viable over the culture period. However, they did not report the MSC conversion toward chondrocyte‐like cells or NP phenotype and did not perform any in vivo tests of that scaffold.

Composites made from PCL and type I collagen or carrageenan gel have shown promise in repairing IVD defects. When cultured under externally imposed loading scenarios, equiaxial loading of a type I collagen‐PCL scaffold seeded with human adipose‐derived MSCs led to 2–4 fold increases in ECM production, matrix fibre reorganisation, cell elongation in the load direction, as well as significant differentiation of MSCs to AF‐like cells (indicated by 30 folds upregulation of AF markers) when compared with unloaded controls [117]. Carrageenan gel‐infused PCL scaffolds have been investigated for their potential to regenerate NP tissue, demonstrating better mechanical properties compared with carrageenan gel alone, an improvement that was attributed to the incorporation of polymeric reinforcements and increased overall material stiffness, comparable to native NP tissue. Preliminary evaluation over 21 days of 3T3 cell culture suggested that the incorporation of these polymeric networks enhanced cellular proliferation compared with the hydrogel alone, suggested to be due to increased access to media and adhesive surface area within the polymer network [164].

Composites of PGA and HA have also been intensively studied [165, 166, 213, 214], confirming their ability to support IVD repair as compared with the control group (nucleotomy only), indicated by greater production of ECM, improved IVD water content, and preservation of IVD height in an ovine lumbar IVDD model [165] and rabbit lumbar IVDD models [166, 213, 214].

#### 3.1.4 Scaffold Properties

While the type of degradable polyester impacts degradation mechanism and rates, and to some extent, processibility and mechanical properties, how these polymers are presented to cells in scaffolds has also been shown to be of relevance for IVD applications. For example, Kim et al. [158] seeded rabbit NP cells onto PLGA scaffolds with various pore sizes and cultured the construct in growth medium. They found that the group with pore sizes of 90–250 μm showed significantly better cell proliferation and ECM production than the other groups, demonstrating the importance of pore size in PLGA scaffolds. Feng et al. [163] reported the regeneration of NP tissue using a biodegradable nanofibrous PLLA scaffold with a pore size of 250–420 μm. When seeded onto the scaffold, rabbit NP cells proliferated faster and produced more ECM (GAG and type II collagen) than those loaded onto solid‐walled PLLA scaffolds. Furthermore, after being implanted into the coccygeal IVD of athymic rats for up to 12 weeks, the biodegradable nanofibrous PLLA scaffold (with rabbit NP cells) behaved similarly to the native NP tissue—indicated by increased ECM deposition and better maintenance of IVD height compared with the solid‐walled PLLA scaffold. Therefore, the architecture used for PLLA scaffolds can be a crucial influencing factor for IVD repair and should be well designed.

### 3.2 Cell Therapy for IVD Repair/Regeneration

From a clinical perspective, the optimal cell sources for IVD regeneration would be autologous NP and AF cells derived from patients, due to host compatibility and lack of immunological issues. However, cell numbers extracted from degenerative IVDs are nominally extremely low and the regenerative potential similarly disappointing [324]. For this reason, most studies have focused on other cell sources as alternatives, particularly adult tissue‐derived stem cell sources that offer accessibility and substantial differentiation potential to form NP‐ or AF‐like cells. The direct application via injection of stem cells alone as a therapy for IVDD is not without its pitfalls, as stable IVD cell phenotypes from differentiated stem cells are necessary endpoints and cell survival in the poor microenvironment of locally degenerated IVDs is questionable [261, 262, 263, 264]. In the next section of this review, the cell sources used in tissue engineering approaches for IVD repair/regeneration are comprehensively described, including stem cells, IVD/AF/NP cells, chondrocytes and fibroblasts. As some growth factors have been used to promote the differentiation of stem cells or stimulate cell proliferation, they are also incorporated in this section.

#### 3.2.1 Stem Cells

**FIGURE 6:**
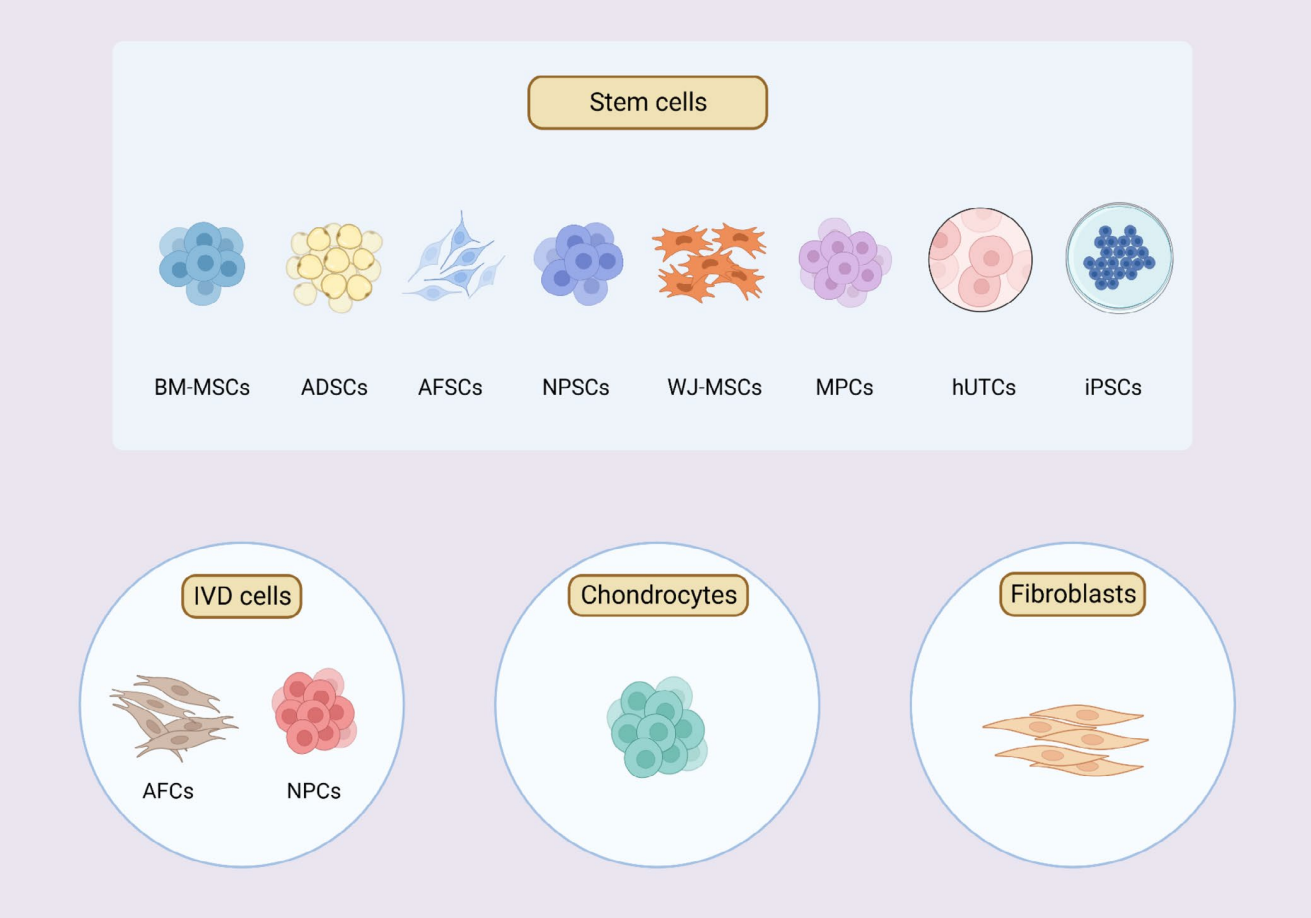
Cells used for engineering IVD regeneration. ADSCs, adipose‐derived stem cells; AFCs, annulus fibrosus cells; AFSCs, AF‐derived stem cells; BM‐MSCs, bone marrow‐derived mesenchymal stem cells; hUTCs, human umbilical tissue‐derived cells; iPSCs, induced pluripotent stem cells; IVD, intervertebral disc; MPCs, mesenchymal progenitor cells; NPCs, nucleus pulposus cells; NPSCs, nucleus pulposus‐derived stem cells; WJ‐MSCs, Wharton's Jelly‐derived mesenchymal stem cells. Created with BioRender.com.

As shown in Figure 6 and Table S1, over the past two decades, a variety of cell sources have been extensively investigated in an attempt to determine the optimal cell source to be incorporated with biomaterials as a therapeutic strategy to treat IVDD‐related diseases—mostly through animal studies or in vitro research. In total, 72 studies have been identified that utilise stem cells in tissue engineering experiments. These stem cells were isolated from different sources, including bone marrow‐derived mesenchymal stem cells (BM‐MSCs), adipose‐derived stem cells (ADSCs), AF‐derived stem cells (AFSCs), NP‐derived stem cells (NPSCs), human umbilical tissue‐derived cells (hUTCs), induced pluripotent stem cells (iPSCs), mesenchymal progenitor cells (MPCs) and Wharton's Jelly‐derived mesenchymal stem cells (WJ‐MSC).

Interestingly, around half of these studies (35/72) employed BM‐MSCs in combination with biomaterials to investigate their repair/regeneration capability. Among them, 6 studies reported autologous cell implantation, 8 studies on allogeneic cell implantation and 11 studies on xenogeneic cell implantation—the others were performed in vitro. Nonetheless, it is difficult to conclude the efficacy of the treatment effect from the different animal species, due to the fact that even though most of the research papers reported positive outcomes, many studies do not compare the therapeutic effect between two stem cell sources using the same animal model, except for a few. Malonzo et al. [167] performed a comparative study between human BM‐MSCs and bovine NP cells in treating ex vivo bovine IVDD models. They found that human BM‐MSCs significantly upregulated gene expression of ACAN, COL2α1, VCAN and SOX9 during culture in the disc cavity, whereas the gene expression profile of NP cells remained unchanged. Henriksson et al. [215] compared human BM‐MSCs with human chondrocytes when loaded in a HA hydrogel. When co‐injected into degenerative porcine IVD induced by puncture injury, they found that both human BM‐MSCs and human chondrocytes could survive at least 6 months in the porcine IVDs, yet the combination of HA gel carrier and mature cells (such as articular chondrocytes) was likely to increase IVD puncture‐caused degeneration instead of promoting regeneration. It is usually necessary to use immunosuppressive drugs in the case of xenotransplantation. However, the animals used in this study were not treated with any immunosuppressors, because IVD can be considered an immunoprivileged zone. Despite this, the authors did not draw a conclusion regarding which of these cell types was the most suitable cell source for IVD repair/regeneration, due to the small sample size that was far from asserting statistical significance. At present, autologous BM‐MSCs are favourable due to their biocompatibility (absence of immune rejection response). Next, allogeneic and xenogeneic cell sources follow behind in terms of preference.

More experiments are however clearly needed to assess the true utility of xenogeneic cell sources, with the advantage of greater availability availing them as a more practical cell source for testing biomaterials, but not suitable for clinical application because of their immunological potential offering a substantial disadvantage. In this review, there are many studies (totally 11) reporting the application of xenogeneic BM‐MSCs for IVD regeneration. In some studies, human BM‐MSCs were seeded in bioscaffolds to form implants and then the implants were transplanted into bovine IVD explants [119, 162, 167, 284], showing effective IVD regeneration, indicative of upregulation of ECM components (ACAN, COL II) and improved biomechanical properties of IVDs. Immune rejection response due to the xenogeneic cell source, however, was not reported because it cannot be observed in ex vivo tests used in those studies. Some other studies underwent in vivo tests [36, 159, 215]. Henriksson et al. [36] encapsulated human BM‐MSCs in a hydrogel carrier which was injected into porcine lumbar IVDD models, indicating that human MSCs can survive in the porcine disc for at least 6 months and express typical chondrocyte markers (ACAN, COL II) suggesting differentiation toward NP‐like cells. Later, the same team co‐injected human BM‐MSCs and HA hydrogel into injured porcine lumbar IVDs, finding that xenotransplanted human MSCs survived in porcine IVDs for 6 months and produced COL II in all six animals, but most MSCs/hydrogel transplanted IVDs showed degenerative changes at MRI and positive bone mineralisation staining [215]. Xiang et al. [159] seeded human BM‐MSCs and NP cells onto a PLGA scaffold and transplanted it into rabbit lumbar IVDD models, finding that the xenogeneic cells‐laden scaffold can maintain the mechanical properties and cell morphology and produce more ECM components (ACAN, COL II) than the group implanted with BM‐MSCs and NPs only. Taken together, all those studies only reported xenogeneic cell sources to regenerate IVDs in vivo, without reports on immunological issues resulting from xenotransplantation.

ADSCs are another promising cell source that has been assessed in combination with bioscaffolds to slow IVDD progression. Altogether, 19 studies have evaluated the potential of ADSCs for treating IVDD animal models, with some of them utilising autologous, allogeneic or xenogeneic models. It was shown that ADSCs can express a NP‐like phenotype (after treatment with the appropriate inducing factors), and help to maintain IVD height, structure and water content [216]. In addition, ADSCs can facilitate native IVD cells to proliferate and upregulate gene expression of ACAN and COL I [217]. In contrast with BM‐MSCs and ADSCs, the remaining cell sources such as the MPCs and iPSCs are far less investigated. To date, MPCs have not been widely used in IVD engineering strategies. MPCs have been reported to possess good biocompatibility and chondrogenic potential in vitro by co‐culture with PPS‐combined HA‐PEG hydrogel [206, 207]. Only one study [218] detected the use of MPCs to repair damaged IVDs in vivo; allogeneic MPCs and PPS were embedded in a gelatin sponge scaffold and then transplanted into an ovine model of lumbar discectomy, the results after 6 months suggesting that the scaffold‐carried MPCs and PPS can result in less reduction in IVD height, maintain better MRI signal and deposit more proteoglycans, compared with either discectomy only or scaffold only groups. Although MPCs have shown the potential of regenerating damaged IVDs, more studies are definitely needed for further clarification.

In the investigation of iPSCs used for IVD engineering, only 2 studies were released, and they are all in vivo studies [168, 219]. Some in vitro studies have demonstrated that iPSCs have the potential of differentiating into NP‐like cells [265, 266] and notochord‐like cells [267, 268]; however, they did not explore the effect of using those iPSCs‐derived cells to repair degenerated IVDs, regardless of in vitro or in vivo. Xia et al. [219] differentiated human iPSCs into NP‐like cells and continuously tested their in vivo ability to regenerate IVDs. They combined gelatin microsphere (for sustained release of GDF‐5) and NP‐like cells and co‐injected them into degenerated rat coccygeal IVDs, showing the regenerative effect of iPSCs‐derived NP‐like cells on IVDs, indicative of restored IVD height and water content and improved ECM deposition compared with the untreated group. Later, Hu et al. [168] evaluated the effects of thermosensitive hydrogels loaded with human iPSCs transfected by GDF‐5 on needle puncture‐induced rat coccygeal IVDD. Three months later, it was found that GDF‐5‐transfected human iPSCs, when combined with hydrogel, can promote IVD regeneration regarding X‐ray, MRI and HE staining scores, as compared with the puncture only and hydrogel only groups. Taking all together, current limited studies indicate the possibility of iPSCs as cell sources for engineering IVD regeneration. While iPSC‐derived NP‐like cells show promise, their tumorigenic potential and epigenetic stability in long‐term studies remain to be clarified. The available information is currently very limited and needs to be explored in the future.

#### 3.2.2 IVD/AF/NP Cells

IVD cells are separately listed as a cell type here because some studies have not split IVD cells into AF and NP cells (it should be mentioned that the term ‘IVD cells’ generally refers to NP cells in the field of spinal research). As shown in Table S1, nearly half of all included studies identified IVD/AF/NP cells as potential cell sources cultured in biomaterials for IVD repair and regeneration regardless of in vivo or in vitro studies. Among them, some studies identified the possible treatment effect of biomaterials encapsulating IVD/AF/NP cells including autologous, allogeneic and xenogeneic cell sources. A typical application of autologous NP cells was performed by Rosenzweig and collaborators [169] who reported the regenerative potential of an autologous NP cell‐seeded HA hydrogel transplanted into human lumbar IVDs isolated from cadavers. Next, the treated IVDs were cultured in a bioreactor under cyclic dynamic loading for 5 weeks. Eventually, the cell‐seeded HA hydrogel showed the potential to regenerate IVD, with improved MRI signal and ECM deposition. Obviously, that study is very clinically relevant to human IVD regeneration by employing a unique human IVD setup, but it would be very difficult to replicate using in vivo models. In another study, autologous bovine NP cells were seeded in a thermo‐reversible hydrogel and then injected into an ex vivo bovine caudal IVDD model cultured under static loading for 8 or 16 days, PCR showing no change of gene expression profile (ACAN, COL2α1, VCAN and COL1α2) [167]. Therefore, the effect of autologous IVD cells on IVD regeneration remains to be further elucidated.

Due to the limited availability of autologous IVD cell sources, most researchers favour allogeneic or xenogeneic cell sources to test the biocompatibility and effectiveness of using new materials for IVD repair and regeneration. Gan et al. [161] transplanted allogeneic rat and porcine NP cells, together with a hydrogel carrier, into degenerated rat tail and porcine lumbar IVDs respectively to test the effectiveness of regenerating IVDs. It was found that the hydrogel‐encapsulated allogeneic rat NP cells can survive in rat tail IVDs for up to 4 weeks while few NP cells can survive in the PBS group because of the immune rejection response to allogeneic NP cells. Also, the hydrogel‐encapsulated allogeneic porcine NP cells can facilitate rehydration and regeneration of porcine degenerative NPs up to 12 weeks, with the encapsulated NP cells showing higher ECM (ACAN and Col II) deposition than the hydrogel‐only group and the cell‐PBS group. In addition to allogeneic cell sources, xenogeneic cell sources have already been investigated in vivo. Human MSCs and NP cells, mixed and encapsulated in a PLGA scaffold, were grafted into degenerated rabbit IVDs, results indicating that the xenogeneic cells in both groups (cells/hydrogel and cells alone) were detectable at 12 weeks post‐surgery, but those carried by a PLGA scaffold produced more proteoglycans and COL II than the control group (cells alone) [159]. Collectively, existing reports suggest that both allogeneic and xenogeneic cell sources can survive after in vivo transplantation and facilitate chondrogenic differentiation to repair degenerated IVDs, but with the precondition of being combined with biomaterials‐based scaffolds. Other studies with IVD/AF/NP cells aimed to verify the biocompatibility and effectiveness of new biomaterials only tested materials using cells without in vivo studies, not applicable (N/A) for discussing cell origins.

#### 3.2.3 Other Cell Sources

It is known that human NP cells are chondrocyte‐like cells, sharing most of their phenotype markers with chondrocytes. Therefore, some researchers have also tried to use chondrocytes as a cell source in IVD repair and regeneration. As exhibited in Table S1, chondrocytes have been tested with biomaterials in 11 studies and only one of them is an animal model test employing a xenogeneic cell source [215]. Briefly, human chondrocytes and a HA hydrogel were co‐injected into injured IVDs of six minipigs. Six months later, the human chondrocytes still survived well and produced type II collagen in all porcine IVDs. However, the chondrocyte/HA hydrogel composite was not successful because of the severe resulting IVDD indicated by worse MRI signals in a third of treated IVDs and positive bone mineralisation staining. In the remaining studies, chondrocytes are usually used to test the biocompatibility of materials in vitro. In addition to chondrocytes, fibroblasts [164, 170] have been utilised in some tests of material biocompatibility but only in vitro, making it difficult to assess the possibility as a cell source for IVD engineering.

#### 3.2.4 Growth Factors and Other Bioactive Molecules

Cytokines and other bioactive molecules are known to play a key role in promoting stem cells to differentiate toward chondrocytes or NP‐like cell phenotypes and facilitate cell growth, proliferation and ECM production. A variety of molecules, including cytokines, have been investigated to determine their effect on stem cell differentiation toward NP‐like phenotypes. These factors are often combined with the bioscaffolds as enhancers for IVD repair/regeneration. As shown in Table 3, bioactive molecules (including growth factors) that have been tested for IVD engineering include TGF‐β1 [53, 120, 171, 269, 270, 345, 346], TGF‐β3 [42, 121, 172, 185, 271], BMP‐2 [171, 285, 347], BMP‐7 [173, 285], GDF‐5 [57, 168, 186, 216, 219, 269, 270, 271], GDF‐6 [187, 271, 343, 344], platelet‐derived growth factor BB (PDGF‐BB) [283], PPS [206, 207, 218], connective tissue growth factor (CTGF) [220], chondroitin sulphate (CS) [188], chemokine (C‐C motif) ligand 5 (CCL5) [122], anti‐miR‐199a [189], stromal cell derived factor‐1 (SDF‐1) [221], fucoidan [123], vasorin [190] and PRP [191]. Generally, 10 ng/mL of TGF‐β1 or TGF‐β3 is contained in the standard differentiation medium used to induce stem cells toward chondrogenesis in vitro. Both TGF‐β1 and BMP‐2 can promote chondrocyte‐like cells to proliferate and produce more GAG in both human and canine models. BMP‐2 caused more type II collagen deposition, but TGF‐β1 increased type I collagen production and fibrotic differentiation [347]. The underlying mechanisms for these results could be that TGF‐β1 induced Smad1 and Smad2 signalling in canines, whereas it only induced Smad2 signalling in humans. BMP‐2 increased Smad1 signalling in both species [347]. Collectively, this indicates that Smad1 signalling was associated with type II collagen production in chondrogenic differentiation of stem cells, whereas Smad2 signalling was associated with fibrotic differentiation.

**TABLE 3:** Bioactive molecules used for engineering IVD regeneration.

Bioactive molecules	In vitro results	In vivo results
TGF‐β1	1. TGF‐β1 promoted rabbit NP cells to produce more aggrecan, COL I and COL II compared with saline control [171]. 2. Human bone marrow‐derived stromal cells cultured with TGF‐β1 caused less KRT‐19 expression and much lower ratio of aggrecan/COL II, compared with GDF‐5 or NP cell culture [269]. 3. TGF‐β1 increased the proliferation and matrix deposition of AF cells in 3D culture and in an ex vivo IVD organ culture model [120].	N/A
TGF‐β1/GDF‐5	TGF‐β1/GDF‐5 can synergistically facilitate the nucleopulpogenic differentiation of the hASCs, as evidenced by NP‐related genes (CD24, OVOS2 and PAX1). The ECM including aggrecan and COL II was upregulated to an equal level of the native NP [270].	N/A
TGF‐β3	1. After 21 days, porcine AF cells (when seeded on porous alginate scaffolds) with TGF‐β3 stimulation produced abundant GAG and COL I, the main ECM in AF [121]. 2. When cultured in TGF‐β3‐supplemented medium for 2 weeks, MSCs with hyaluronan hydrogel produced more GAG and COL II than the basic medium only group [172]. 3. Compared with other groups, the group with TGF‐β3 produced more aggrecan, SOX9, COL II and COL X [185].	TGF‐β3 can help to drive hBMSCs toward chondrogenic differentiation, facilitating IVD regeneration in a beagle dog model [185].
BMP‐2	Compared with the saline control group, BMP‐2 can stimulate rabbit NP cells to produce aggrecan, COL I and COL II [171].	BMP‐2 or BMP‐2/7, bound tofibrin/HA hydrogel, was injected into slightly degenerated IVD in a goat model. After 12 weeks post‐surgery, the bound BMP‐2 and BMP‐2/7 were safe to the animals. However, IVD regeneration was not detected in terms of the MRI T2‐weighted signals in NP tissues, GAG deposition, collagen content (hydroxyproline) and histological grading [285].
BMP‐7	BMP‐7‐conjugated hydrogel drive human NP cells to show higher proliferation and produce more COL II, Sox‐9 and aggrecan but less COL I after 28 days compared with control [173].	N/A
GDF‐5	1. Sustained release of GDF‐5 promoted human stem cells differentiation to an NP‐like phenotype [216]. 2. Human BM‐MSCs were cultured in TGF‐β1 or GDF‐5 supplemented medium. The hypoxic GDF‐5 group produced most aggrecan and COL II mRNA levels and GAG accumulation, as well as highest expression of NP markers CA12 and KRT‐19 [57]. 3. GDF‐5‐GelMa up‐regulated the expression of NP‐like phenotype markers, including COL2, ACAN, KRT19 and CD24 after 2–3 weeks' culture [186].	1. Thermosensitive hydrogels encapsulating hiPSCs overexpressing GDF‐5 showed better repair effect compared with the hydrogel alone group in a rat IVD degeneration model, in terms of X‐ray, MRI and HE staining scores [168]. 2. In a rat IVD degeneration model, the administration of GDF‐5 slowed the descent speed of IVD height, water content and NP structure, compared with other treatment groups [216]. 3. GDF‐5 could reverse IVD degeneration in a rat model, showing increased water content, NP cell number and ECM, as well as recovered IVD height [219]. 4. The GDF‐5‐GelMa group showed a better IVD structure and morphology, with the highest DHI among all groups [186].
GDF‐6	1. When cultured in differentiating media, GDF‐6 induced human MSCs to upregulate gene expression of NP markers including a higher aggrecan/COL II ratio and produce more sGAG, compared with the effects of TGF‐β3 or GDF‐5 [271]. 2. PLGA/PEG microparticles‐delivered GDF‐6 effectively induced hASCs to upregulate NP marker gene expression and increase GAG and aggrecan production regardless of normoxia or hypoxia, compared with controls [343]. 3. The soluble GDF‐6, combined with PNIPAAM‐g‐CS and alginate, facilitated adipose derived mesenchymal stem cells to differentiate toward NP‐like phenotypes [187].	After being injected in an ovine IVD model, GDF‐6 can attenuate the loss of IVD height and ECM proteins (proteoglycan and COL II), maintain greater hydration and possess more cells in the NP [344].
PDGF‐BB	In 3D pellet culture, the chondrogenic markers aggrecan, COL I, COL II and Sox9 was produced far less in human MSCs cultured with platelet‐rich plasma compared with TGF‐β1, at mRNA and protein level [345].	Used in a rabbit IVD degeneration model, PDGF‐BB was found to decrease cell apoptosis and deposition of COL III in NP&AF and cause higher MRI signal of injured IVDs, less degeneration indicated by degenerative scores, fewer indicators of degeneration by biomechanical assessments and higher compressive strength to failure [283].
PPS	PPS with hydrogel caused more GAG and COL II deposition than the hydrogel alone [206]. Compared with soluble PPS, bound PPS (HA‐PPS) promoted more production of GAG and collagens [207].	In an ovine microdiscectomy model, compared with scaffold‐only group, PPS and MPCs embedded in a gelatin/fibrin scaffold resulted in significantly less reduction in IVD height, better MRI signal, more NP proteoglycan content and improved IVD morphology [218].
CTGF/TGF‐β3	CTGF and TGF‐β3 induced BM‐MSCs to differentiate into NP‐like cells and AF‐like cells [220].	N/A
Chondroitin sulphate (CS)	Higher amounts of CS in the gel composition increased the production of sulphated GAG and COL II [188].	N/A
CCL5	When cultured with AF cells, CCL5 did not change gene expression of CCL5 receptors, catabolic and proinflammatory markers [122].	Tested with an organ culture model, CCL5 did not drive AF cells toward the defect sites. CCL5, when used in sheep models, did not demonstrate any repair effect on the AF [122].
Anti‐miR‐199a	MSC/HP‐anti‐miR‐199a/NS/NF‐SMS constructs were found to facilitate NP phenotype in vitro [189].	When injected into a rabbit lumbar degeneration model, MSC/HP‐anti‐miR‐199a/NS/NF‐SMS can cause the synthesis of extracellular matrix, maintain IVD height and prevent IVD calcification [189].
SDF‐1	N/A	Although HA/SDF‐1 hydrogel resulted in higher cell number, no IVD regeneration was found [221].
Fucoidan	Fucoidan inhibits LPS‐induced inflammatory and oxidative stress in AFCs [123].	Fucoidan‐loaded nanofibrous scaffolds promoted rat AF repair by ameliorating the harsh degenerative microenvironment [123].
Vasorin	Vasorin protein enriched in EVs promoted the proliferation and ECM anabolism of NP cells via the Notch1 signalling pathway [190].	Vasorin‐loaded decellularised ECM hydrogel can efficiently slow IVD degeneration and achieve better therapeutic effects in a rat tail model [190].
PRP	N/A	PRP promotes NP regeneration in a rat coccygeal AF puncture model [191].

Abbreviations: AF, annulus fibrosus; AFC, annulus fibrosus cell; BM‐MSCs, bone marrow‐derived mesenchymal stem cells; BMP, bone morphogenetic protein; CA12, carbonic anhydrase 12; CCL5, chemokine (C–C motif) ligand 5; COL, collagen; CTGF, connective tissue growth factor; DMEM, Dulbecco's modified eagle medium; ECM, extracellular matrix; GAG, glycosaminoglycan; GDF, growth differentiation factor; HA, hyaluronic acid; hASCs, human adipose stromal cells; hiPSCs, human‐induced pluripotent stem cells; IVD, intervertebral disc; KRT‐19, keratin‐19; MPCs, mesenchymal precursor cells; NP, nucleus pulposus; OVOS2, ovostatin 2; PAX1, paired box 1; PDGF‐BB, platelet‐derived growth factor BB; PEG, polyethylene glycol; PLGA, poly(dl‐lactic acid‐*co*‐glycolic acid); PPS, pentosan polysulphate; PRP, platelet‐rich plasma; SDF‐1, stromal cell derived factor‐1; TGF, transforming growth factor.

In a large animal study, the BMP‐2 and BMP‐2/7 were included in a fibrin/HA‐hydrogel, showing no effect on IVD regeneration [285], in contrast to another report indicating that BMP‐7 short motif‐designed functional self‐assembling peptide nanofiber hydrogel was an excellent scaffold that can better promote IVD tissue repair [173]. Compared with TGF‐β1, GDF‐5 resulted in more expression of NP markers KRT19 [57, 269] and CA12 [57] and a higher ratio of aggrecan/COL II gene expression [269] when used alone to promote nucleopulpogenic differentiation of human MSCs. Interestingly, TGF‐β1 and GDF‐5 can exert a synergistic effect on human ADSCs differentiation toward NP‐like phenotype, the mechanism being that Smad1/5/8 and Smad2/3 were the main downstream effectors that were activated in human ADSCs upon TGF‐β1 and GDF‐5 stimulation. The Smad2/3 pathway mainly governed the acquisition of the NP cell molecular phenotype while the Smad1/5/8 pathway controlled the NP cell morphology [270]. Compared with TGF‐β3 and GDF‐5, GDF‐6 was a more suitable growth factor because it can induce human MSCs to express more NP markers (KRT8, KRT18, KRT19 and brachyury T), generate higher aggrecan/COL II ratio and deposit more GAG [271]. PPS, not a growth factor, but a synthetic and soluble GAG‐like factor, has been tested for IVD engineering [206, 207, 218]. It has been used to induce chondrogenesis in human MPCs, as evidenced by more proteoglycan and COL II deposition but less COL I [206, 348]. Moreover, a bound PPS to HA (forming HA‐PPS) can work better when incorporated into PEG‐HA hydrogel, driving human MPCs to produce more GAG and COL II than the soluble PPS [207]. Furthermore, the effect of PPS on IVD repair has been verified in vivo using an ovine model [218].

### 3.3 Animal Models Used for Engineering IVD Regeneration

**FIGURE 7:**
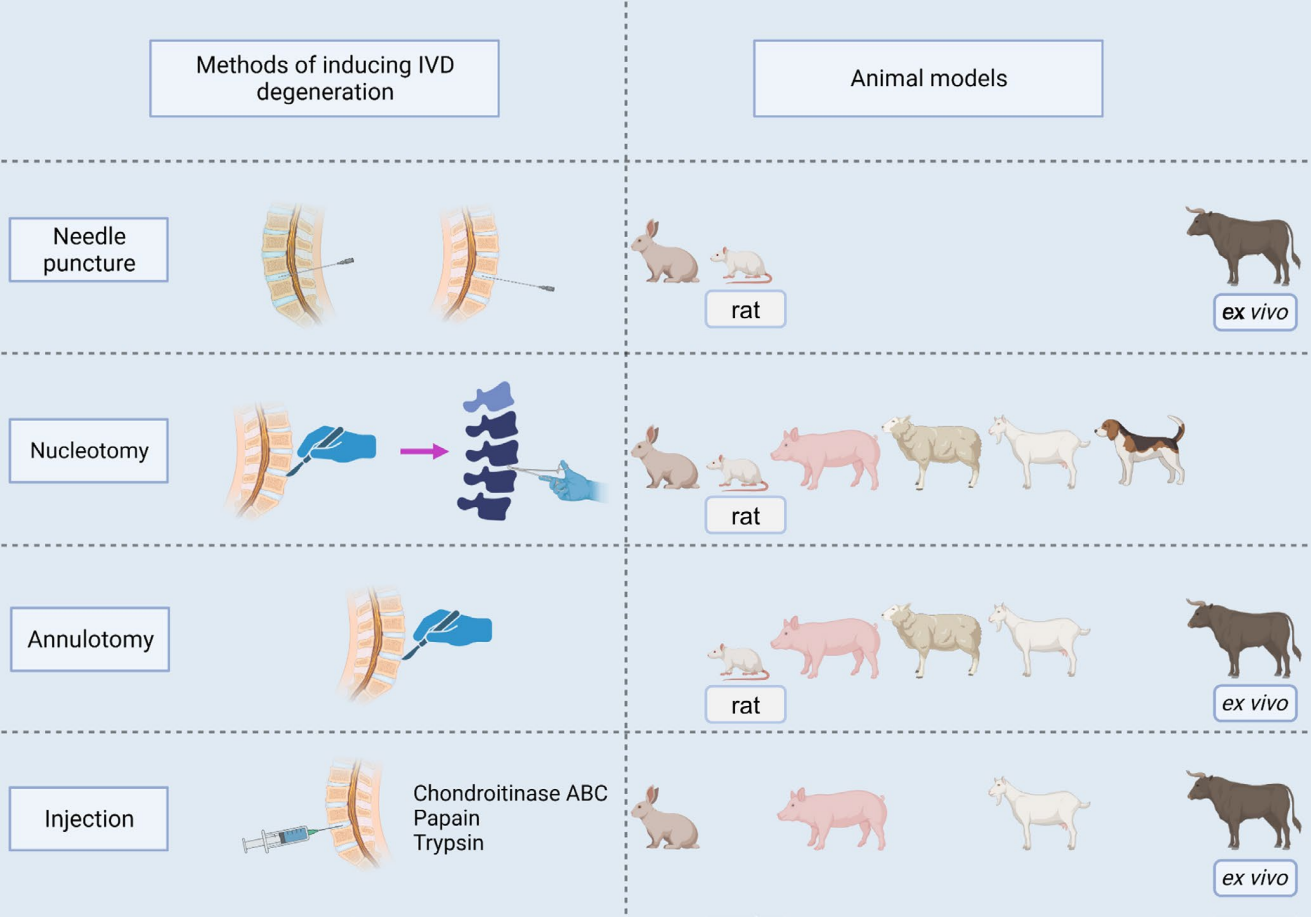
Animal models used for engineering IVD regeneration. Created with BioRender.com.

IVDD animal models have been and are being widely used to investigate the effects of tissue engineering strategies on IVD regeneration. Thus, a good understanding of current IVDD animal models would benefit all IVD engineering research. In the past decades, a variety of stable and reliable animal models have been established and verified, most aiming to mimic age‐related IVDD progression and outcome in humans. These animal models include large, medium and small animal models, such as sheep, rabbit and rat models, respectively. As shown in detail in Figure 7 and Table S2, large animal models consist of porcine, ovine, bovine, canine and caprine IVDD models; medium models here refer to rabbit models which are the most commonly used IVDD models in all in vivo experiments; the small animal model here is the rat model as mice are not suitable for testing biomaterials and cell source due to the limited IVD space. There are different advantages for each model. In general, the advantage of using a small animal model is that the animals are cheaper to buy, maintain and easier to handle; the downside is that the injection volume into the IVD space is quite limited and biomechanical analysis is more difficult and less relevant compared with large animal models [216].

So far, IVDD models have been well established using various approaches including physical and biochemical methods; physical ways are comprised of AF puncture, nucleotomy (including total and partial resection), annulotomy and TDR, while biochemical methods are composed of chondroitinase ABC (in vivo) and papain‐ or trypsin‐induced IVDD models (ex vivo) [349]. Among all studies included, the physical approaches (AF puncture and nucleotomy) are the most popular methods used to establish IVDD models for IVD tissue engineering. In addition to the aforementioned approaches to induce IVDD models, some other methods have also been set up, such as IVDD models induced by IVD instability [349, 350], ovariectomy (OVX model) [349, 351, 352] and IVD injection of AGE (advanced glycation end‐products) [353, 354, 355]. However, all of these IVDD models are for small animals (rats and mice) and have not previously been used to test biomaterials.

#### 3.3.1 Rat Model

Among all species, the rat IVDD model is the most commonly used model for IVD tissue engineering. In total, 33 studies using rat models are included in this review. All studies used a rat tail IVDD model. Unlike the rabbit models, the injection volume in the IVD space of an adult rat (3 months old) is quite limited. As reported in the literature, only 2–15 μL can be injected into the IVD space [168, 174, 175]. The injection process can take up to 5 min in case of any leakage [176]. The rat tail IVDD models can be induced by various approaches, AF puncture [168], AF defect [124] and nucleotomy [217] in a posterolateral way.

Currently, there are two surgical procedures used to induce the IVDD rat model; one is through an open surgery with incision, the other is minimally invasive by using fluoroscopy guidance. Feng et al. [163] performed the IVD surgery in 3‐month old rats under direct vision. They made a longitudinal incision (3‐cm long) to expose the native disc, then removed the NP entirely. Following this, they inserted a tailored scaffold with NP‐like shape (1.0 mm in height, 2.5 mm in diameter) loaded with 9 μL of cell suspension (3 × 10^7^ NP cells/mL) into the IVD space. Gan et al. [161] performed a similar exposure surgery to stab the AF and aspirate out the NP using a 19‐gauge needle. NP cells were loaded onto the hydrogel at a density of 10^7^/mL and 10 μL was injected into the tail disc. Alternatively, AF puncture surgery can be conducted under fluoroscopy guidance without an incision. Parallel to the CEP, a 21‐gauge needle is vertically inserted into the centre of an IVD under fluoroscopy guidance, at a depth of 5 mm from the skin, followed by 180° of rotation and a 5‐s hold [174]. In contrast, in most studies the needle would be rotated 360° and held for 30 s [177, 216, 219, 222]. Nonetheless, all studies reported obvious degeneration of the disc despite small differences in the methods used. In another study, Than et al. [210] used palpation to locate the Co5/Co6 segment, defined as the level below the last palpable transverse process. They found that the Co5/Co6 level could be precisely located 2 cm from a rat's anus. A 21‐gauge needle puncture was performed to cause AF injury with live fluoroscopy guidance used to keep the needle tip parallel to the CEP and in the centre of NP.

#### 3.3.2 Rabbit Model

The rabbit IVDD model is the second most popular model used for investigating the combined effect of cell‐laden biomaterials. The injection volume in the IVD space of an adult rabbit can be 100–300 μL [178, 223, 224, 356]. In total, 10^7^ cells/mL can be injected into the IVD space using a 27‐gauge needle [356]. The rabbit IVDD model can be developed in a few different ways, including AF puncture, nucleotomy and a combination of the two.

Nucleotomy is the most commonly used method to cause rabbit IVDD and was performed in nine studies excluding the combination method [125, 126, 159, 166, 179, 192, 213, 214, 224, 225]. The results demonstrated that nucleotomy is a reliable approach that can lead to an easily reproducible IVDD rabbit model. In addition, AF puncture is used to induce IVDD rabbit models, having shown similar reliability and reproducibility as nucleotomy [151, 155, 178, 180, 189, 193, 209, 283, 289]. An alternative way to cause IVDD in rabbits is a combination of the two approaches [223, 226, 227, 228]. This was shown to be a more efficient method to develop IVDD due to the synergetic effects between the two procedures [223]. Nakashima et al. [223] performed IVD puncture in 6 rabbits (24 discs) and IVD puncture plus NP aspiration in 3 rabbits (12 discs) using 23‐gauge needles via an abdominal opening approach. One month later, only one third of injured discs (8 discs) in the IVD puncture group progressed to degeneration, indicated by a decrease in signal intensity on T2‐weighted MRI, and 2 IVDs recovered back to the initial level at 2 months post‐surgery. By contrast, all injured IVDs in the puncture/aspiration group degenerated, indicated by decreased T2‐weighted MRI signal intensity at 1 month post‐surgery and none of the degenerated IVDs showed any evidence of recovery at 2 months post‐surgery.

AF puncture alone can successfully induce IVDD models in rabbits. A range of needle sizes can be selected for AF puncture‐induced models, including 16‐gauge [155], 18‐gauge [178, 283] and 21‐gauge needles [151, 227], with the exception of 23‐gauge needles (which only successfully induced 1/3 IVDD) [223]. In the combined method, a thinner needle is generally used to stab IVDs, such as 23‐gauge [223] and 21‐gauge [226, 228, 356] needles, before aspirating the NP. Relative to the puncture needles, the injection needles (such as 27‐gauge needles) used to deliver the cell‐laden hydrogels into the IVD space are much narrower [356]. In general, with consideration to the needle dimensions, most of the AF puncture surgeries are performed via an anterior retroperitoneal pathway, with a 5 mm‐puncture depth required.

Additionally, some other surgical procedures have been reported. Subhan et al. [180] used an 18‐gauge needle to carry out AF puncture via a left posterolateral approach, which was 2.5 cm from the midline spinous process at a 30°–45° angle from midline to lateral, keeping parallel with the CEP without causing damage. The NP was then injured by wobbling the puncture needle five times in the centre of NP. Also, Ahn et al. [226] inserted a 21‐gauge needle percutaneously into the centre of IVDs via a posterolateral approach under the C‐arm fluoroscopy, and the NP was aspirated using a 10‐mL syringe. So et al. [209] utilised a posterolateral approach to perform the IVDD surgery, and the transverse processes of the vertebrae were resected to expose one side of the target IVDs. The disc was then pierced using a 2.0‐mm wire to cause IVDD. Sato et al. [125, 126] used a laser to vaporise the NP to generate IVDD models. Sawamura et al. [224] and Nagae et al. [225] induced IVDD models by partially aspirating 0.005–0.008 g of the NP, but such a method may be limited by the weight of the NP.

#### 3.3.3 Porcine Model

There have been 7 studies that use a porcine IVDD model. Most of the models were produced by open surgery and some were generated by minimally invasive methods. Compared with the small animal models, the pig models allow a higher injection volume, up to 1 mL [181]. Henriksson et al. [36, 215] employed minipigs (10–15 kg) to perform open spinal surgeries under direct vision. They made a 5 cm incision at the paravertebral site to expose the disc of interest and then aspirated the NP using a 20‐gauge needle. Kang et al. [112] performed open surgeries to expose the IVDs (L2–L6) of the pigs (50 kg) and then produced a model of AF defect for scaffold implantation.

In addition to the injury‐induced models, the IVDD pig model can be induced via injection of chondroitinase ABC into the NP. It was reported that 200 μL of 0.5 U/mL chondroitinase ABC can develop a satisfying degree of IVDD within 4 weeks post‐surgery [182]. The injection is minimally invasive compared with the aforementioned open surgery, but it must be carried out under fluoroscopic guidance. Omlor et al. [181] surgically exposed the lumbar IVDs of pig models via an anterolateral retroperitoneal approach and performed AF puncture combined with partial nucleotomy (0.06–0.14 g, ~10% of NP) using a 16‐gauge cannula with an interior needle. The NP can be aspirated using the syringe, which can be later used to inject 1 mL of cell‐laden hydrogel into the IVD through the cannula.

#### 3.3.4 Ovine Model

Ovine model is one of the common large models used for in vivo tests of some materials designed for IVD repair and regeneration. The advantages of this model are that it allows for biomechanical tests, a higher injection volume of cell‐laden hydrogels, as well as solid construct implantation. Large IVDs can provide more disc space for researchers to operate with. In total, there are 8 studies reporting the ovine‐based IVDD models used for biomaterial tests. All studies share some common surgical procedures. The sheep undergo a surgery in an anterolateral way, with most being open surgeries. Only one study reported that a dorsal endoscopic approach was used to perform partial nucleotomy [165]. All IVDD models were produced on the lumbar IVDs, except one model of cervical AF defect [122]. The surgical methods used to cause IVDD include annulotomy and nucleotomy. Gluais et al. [113] employed female sheep (1 year old, around 30 kg) and used a lateral retroperitoneal approach to perform a box annulotomy (2 × 5 mm) in the outer AF to produce AF defect models. Similarly, Ledet et al. [111] carried out the AF defect surgeries to produce IVDD models using the aforementioned procedures, but instead used male sheep in their study. Twenty‐six weeks postoperatively, those male sheep showed clear IVDD indicated by decreased MRI signal and biomechanics, and worse histological changes. In another study [218], the same surgical procedures were applied, but instead, the researchers used a combination of annulotomy and nucleotomy—successfully establishing an ovine IVDD model.

Some other studies reported different approaches to produce an IVDD ovine model, but they have not been used for testing biomaterials. Lim et al. [357] reported a new IVDD‐producing method using a drill bit to injure the IVDs and found that it successfully caused IVDD. Subsequently, the same group [358] compared two approaches to produce IVDD models; one was a drill bit‐injured model, and the other was induced by a combination of annulotomy and nucleotomy. Compared with the drill bit‐injured model, the annulotomy plus nucleotomy can better reproduce the pathobiological changes that human beings would experience after discectomy, indicating that the latter approach is more appropriate for inducing IVDD in ovine models. Schwan et al. [359, 360] performed a minimally invasive surgery to produce IVDD in ovine models via a posterolateral way. Under X‐ray guidance, they firstly created a surgical corridor to the NP using an awl, and then put a rongeur into the corridor to partially remove the NP tissue. This type of surgery is more difficult than those previously mentioned, as placing a rongeur inside the corridor created by the awl is a tricky procedure. Another approach to induce IVDD in an ovine model was the ventral surgical approach which has been investigated in cervical spines [361]. It showed that cervical IVDs had easier surgical access relative to lumbar spines, and the cervical IVD height is higher as well. Thus, the anterior cervical approach might be a more appropriate way to produce IVDD in ovine models, yet most ovine models are currently based on lumbar spine IVDs.

#### 3.3.5 Caprine Model

There are 4 studies which use caprine models to test biomaterials for IVD tissue engineering in this review. The advantages of using caprine models are similar to those of ovine models. The surgical approaches to induce IVDD include chondroitinase ABC, annulotomy and nucleotomy. Gullbrand et al. [362] compared two approaches to induce IVDD in caprine models; one was to inject chondroitinase ABC into the IVDs, and the other was to do a partial nucleotomy. They surgically exposed the IVDs via a lateral retroperitoneal transpsoatic approach. It was found that both the injection group and the nucleotomy group progressed to IVDD, indicated by MRI and histological changes 12 weeks postoperatively. The same research group then tested a novel type of hydrogel designed for IVD regeneration using the caprine models induced by injection of chondroitinase ABC [183]. Moreover, in another study, they implanted a tissue‐engineered artificial IVD into the cervical spine of the adult goats after partially removing AF and NP tissue via an anterior surgical approach [212]. Peeters et al. [285] tested their hydrogel in caprine IVDD models caused by chondroitinase ABC, which resulted in mild degeneration. Xu et al. [127] also conducted an open surgery in 6‐month‐old male goats to expose the IVDs and created an AF defect (1 cm × 1 cm) to test the repairing effect of an implant consisting of gelatin sponge, MSCs and platelet‐rich plasma.

#### 3.3.6 Bovine Model

Bovine models were employed by only 3 studies to investigate the effect of biomaterials on IVD repair and regeneration [184, 229, 284]. One possible reason could be that the animals are too large and therefore unsuitable to be used in in vivo tests. Furthermore, the cost of maintenance, handling, anaesthesia, and surgery is far greater compared with using a smaller model. Nonetheless, bovine models are still used as ex vivo FSU organ models, which can be helpful in bridging the gap between in vivo and in vitro experiments. There are a number of advantages of using these models [229]. Firstly, biomechanical tests can be easily performed using these models due to the IVD volume. Secondly, the ex vivo FSU organs are suitable for assessing biomaterial implantation and AF defect closure methods. Finally, the tissue response can be tested with these models. For instance, the ex vivo bovine models were previously used for biomechanical testing of IVDs after bioscaffold implantation in two different studies [184, 229]. In addition, IVDD models can be established by needle (21 gauge) puncture and proinflammatory factors using ex vivo organ culture. This is a suitable approach used to test novel therapeutic strategies, as well as cellular and molecular mechanisms [284]. Hence, using ex vivo IVD models can, to some extent, reduce the necessity of some in vivo experiments.

#### 3.3.7 Canine Model

Canine models were reported in 4 studies investigating the effect of biomaterials on IVD repair [160, 185, 230, 363]. IVDD models used in these studies were produced by partial nucleotomy, as reported in previous publications [364, 365]. Ruan et al. [160] constructed a composite based on a PLGA scaffold encapsulating NP cells and investigated the effect on IVD repair using a canine IVDD model successfully established by nucleotomy. Bach et al. [363] transplanted porcine notochordal cells‐derived matrix into the canine IVDs for treatment after using nucleotomy to produce the IVDD model. Later on, another study [230] evaluating the IVD regeneration effect of MSCs encapsulated in PEGDA‐microcryogel also employed the canine IVDD models, which were also successfully developed using nucleotomy.

#### 3.3.8 Differences in Animal Models

When using an animal model in IVD studies, there are some differences between animals that should be considered, such as biomechanics and sex differences. Monaco et al. [366] performed a comparative study in terms of IVD biomechanical properties among bovine, ovine and porcine models, and found that bovine tail lamellae were the strongest and stiffest (in tension), while ovine lumbar lamellae were the weakest and most compliant. Bovine lamellae also had the greatest proportion of collagen, which increased the strength and stiffness. Newell et al. [367] used bovine motion segments to test the mechanical function of the NP under high loading rates and found that the NP pressure did not affect the load transfer through the IVD or the energy it absorbed. The AF might play a more important role than the NP in transferring load and absorbing energy at high rates. Nuckley et al. [368] studied the biomechanical characteristics using an aged IVDD primate model, where they found increased stiffness and decreased energy absorption at the advanced stage of IVDD.

These associations linking the dynamics of the IVD and its degeneration degree are similar to those found in humans. Clinically, LBP and spinal impairments are more commonly seen in women than men, and the underlying mechanical mechanisms were explored by Mosley et al. [369], who utilised rat IVD puncture models to investigate the sex differences in IVD structure and function—finding that both males and females progressed to IVDD after AF puncture, but the degeneration grade in the AF region was higher in females than in males. Moreover, biomechanical tests indicated that female IVDs showed less torsional stiffness, torque range and viscoelastic creep responses in outer AF, as compared with males. Taken together, there may be a sex difference in the biomechanics of IVDs, which remains to be investigated in the future.

## 4 Conclusions and Future Outlook

IVDD is a common and chronic process, eventually causing severe spinal issues. It is not uncommon in clinical scenarios for patients to suffer from LBP and leg pain, both of which result from long‐term IVDD. A typical surgical solution for this issue is discectomy followed by intervertebral fusion to decompress the nerve roots and restore spinal stability. However, this strategy often causes the progression of adjacent segment degeneration because of the changed biomechanical conditions. In the long run, this current therapeutic strategy is not an optimal option for patients. Therefore, it holds promise that bioengineering approaches in regenerating the IVD structure could help to restore its biomechanical function and provide a sound alternative to traditional surgeries.

In pursuit of an optimal approach, bioscaffolds are made from a wide variety of materials and polymers that have been extensively investigated to date. Some researchers utilised bioscaffolds alone while others combined them with exogenous cells (stem cells, nucleus pulposus cells, etc.), the latter having more success. Most of the bioscaffolds are hydrogels, injectable and biodegradable. All the materials used to fabricate bioscaffolds are polymers, either natural, synthetic or a combination of the two. Generally, the advantages of natural polymers for IVD tissue engineering are higher biodegradability, renewability and low/non‐toxicity, while synthetic polymers possess better tailorability, higher mechanical strength and conductivity, as well as a controllable degradation rate. Hence, incorporating all advantages of natural and synthetic polymers into composite materials can improve the scaffolds' properties. The most used natural polymer is hyaluronic acid while the most popular synthetic polymers are PCL. Meanwhile, PLLA, PLGA, PEG and PGA are also commonly used synthetic polymers. When natural/synthetic composites are fabricated, PEG is the most used synthetic polymer combining with HA. To the best of our knowledge, there is no consensus as to which synthetic or natural polymer is the best option for the fabrication of scaffolds/hydrogels. Nevertheless, combining synthetic and natural polymers together to improve the mechanical properties of the scaffold is the most promising strategy for regenerating the IVD to date. For instance, as an efficient inherent antibacterial material, quaternised chitosan can be incorporated into hydrogel to reduce potential infection incidence [370]. Another useful strategy to improve the function of scaffolds/hydrogels is optimising the fabrication and chemical structure. For example, PPS can be covalently bound to HA forming HA‐PPS which, compared with soluble PPS, can better promote MPCs to differentiate into chondrocytes in PEG/HA‐based hydrogels [207].

Although a variety of cell sources have been widely screened, consensus on the best cells used for IVD tissue engineering has not been reached. Nonetheless, the prevailing view on the optimal cell sources suitable for clinical treatment is NP cells from the patients themselves. However, a great drawback is that the cells derived from the patient IVDs are few in number, and the regeneration potential is disappointingly low. Thus, the stem cell sources from the patients might be a better choice due to their availability and the differentiation potential to NP‐like cells—although the application of stem cells as a clinical therapy for IVDD still requires more thorough investigation.

Finally, animal models that mimic IVDD are crucial platforms used to assess the potential of bioscaffolds and cell implantation for IVD repair and regeneration. Due to the specific biomechanical structure of human beings, there are no animal models that are able to completely mimic the IVDD of humans. From the literature, the most investigated IVDD models are rats as they are appropriate in size and are easier to access, handle and manage than larger animal models.

In conclusion, IVD tissue engineering provides a promising approach to regenerate the native IVD structure and restore its function. However, the biomaterials and cell sources used for IVD tissue engineering are numerous, with no consensus reached. Thus, further studies should try to confirm the optimal combination of bioscaffold and cells.

## Author Contributions

**Sidong Yang:** conceptualisation, resources, writing – original draft, review and editing. **Farhad Soheilmoghaddam:** supervision, resources, writing – review and editing. **Peter Pivonka:** supervision, writing – review and editing. **Joan Li:** supervision, writing – review and editing. **Samuel Rudd:** writing – review and editing. **Trifanny Yeo:** writing – review and editing. **Ji Tu:** writing – review and editing. **Yibo Zhu:** resources. **Justin J. Cooper‐White:** conceptualisation, supervision, writing – review and editing, funding.

## Ethics Statement

The authors have nothing to report.

## Conflicts of Interest

The authors declare no conflicts of interest.

## Supporting information


**Table S1.** Cells encapsulated in biomaterials for IVD repair and regeneration.
**Table S2.** Animal models used for engineering IVD regeneration.

## Data Availability

Data sharing is not applicable to this article as no new data were created or analyzed in this study.
