# Supplementary material for: Engineering Intervertebral Disc Regeneration: Biomaterials, Cell Sources and Animal Models
Source: Cell Prolif. 2025 May 19;58(9):e70046. doi: 10.1111/cpr.70046 (PMC12414645; doi:10.1111/cpr.70046)
Supplement: Supplementary file 1 — Table S1. Cells encapsulated in biomaterials for IVD repair and regeneration. Table S2. Animal models used for engineering IVD regeneration. [file CPR-58-e70046-s001.pdf]

Table S1 Cells encapsulated in biomaterials for IVD repair and regeneration.

In total	Cells	Autologous	Allogeneic	Xenogeneic	In vitro only
72	**Stem cells**				
35	BM-MSCs	[112][181][305][194][227][228]	[222][183][127][180][193][192][189][221]	[162][159][284][167][119][215][36][242][201][145][185]	[278][135][172][292][304][53][236][233][220][273]
19	ADSCs	[230] [240] [241]	[217][155] [177] [196] [186]	[216] [187]	[152] [117] [149] [281] [237] [287] [296] [143] [204]
2	AFSCs	--	--	--	[142] [135]
4	hMSCs	--	--	[151] [198]	[153,306]
2	WJ-MSCs	--	--	[226] [199]	--
1	NP-MSCs	--	[176]	--	--
3	MPCs	--	[218]	--	[206,207]
1	hUTCs	--	--	[289]	--
2	iPSCs	--	--	[219,168]	--
3	**IVD cells**	--	--	[178]	[211] [311]
27	**AF cells**	--	[124] [125]	[212,175] [122] [120]	[132][138][131,137][234][110][238][121][208] [302] [116][232][139][205][115,126][143][118][275][123][148]
44	**NP cells**	[169] [167]	[183][161][179][154,160][195][197]	[212][174][159][163][175,178][198]	[215][277][158][234][280][282][156][237][286] [173][208][302][290][171][291][150][294][157] [293][298][299][205][300][301][200][191][274][190]
11	**Chondrocytes**	--	--	[215]	[279][272][128][156][172][129][232][295][141][188]
2	**Fibroblasts**	--	--	--	[170] [164]

ADSCs, adipose-derived stem cells; AF, annulus fibrosus; AFSCs, AF-derived stem cells; BM, bone marrow; BM-MSCs, bone marrow-derived mesenchymal stem cells; hMSCs, human mesenchymal stem cells; hUTCs, human umbilical tissue-derived cells; iPSCs, induced pluripotent stem cells; IVD, intervertebral disc; MPCs, mesenchymal progenitor cells; N/A, not applicable; NP, nucleus pulposus; WJ-MSC, Wharton's Jelly-derived mesenchymal stem cells

Table S2 Animal models used for engineering IVD regeneration.

Species	Study ID	Total studies
Rat	[212,216,217,219,175,124,222] [177] [174] [161] [134] [233] [231] [210] [163] [168,176] [196] [242] [239] [186] [145] [199] [144] [197] [221] [191][273][204] [275][123] [190] [148]	33
Rabbit	[151] [155] [159] [283] [226] [180] [166] [289] [179] [178] [213] [223] [224] [214] [209] [225] [227] [126] [228] [125] [154] [194] [193] [192] [189]	25
Porcine	[112,181] [215] [182] [305] [36] [141]	7
Ovine	[113] [165] [218] [111] [198] [240] [241] [122]	8
Caprine	[212] [183] [127] [285]	4
Bovine	[229] [284] [184]	3
Canine	[160,363,230] [185]	4

IVD, intervertebral disc; IVDD, intervertebral disc degeneration
